# Pharmacotherapy, alternative and adjunctive therapies for eating disorders: findings from a rapid review

**DOI:** 10.1186/s40337-023-00833-9

**Published:** 2023-07-06

**Authors:** Sarah-Catherine Rodan, Emma Bryant, Anvi Le, Danielle Maloney, Stephen Touyz, Iain S. McGregor, Sarah Maguire, National Eating Disorder Research Consortium, Phillip Aouad, Sarah Barakat, Robert Boakes, Leah Brennan, Emma Bryant, Susan Byrne, Belinda Caldwell, Shannon Calvert, Bronny Carroll, David Castle, Ian Caterson, Belinda Chelius, Lyn Chiem, Simon Clarke, Janet Conti, Lexi Crouch, Genevieve Dammery, Natasha Dzajkovski, Jasmine Fardouly, John Feneley, Amber-Marie Firriolo, Nasim Foroughi, Mathew Fuller-Tyszkiewicz, Anthea Fursland, Veronica Gonzalez-Arce, Bethanie Gouldthorp, Kelly Griffin, Scott Griffiths, Ashlea Hambleton, Amy Hannigan, Mel Hart, Susan Hart, Phillipa Hay, Ian Hickie, Francis Kay-Lambkin, Ross King, Michael Kohn, Eyza Koreshe, Isabel Krug, Jake Linardon, Randall Long, Amanda Long, Sloane Madden, Sarah Maguire, Danielle Maloney, Peta Marks, Sian McLean, Thy Meddick, Jane Miskovic-Wheatley, Deborah Mitchison, Richard O’Kearney, Shu Hwa Ong, Roger Paterson, Susan Paxton, Melissa Pehlivan, Genevieve Pepin, Andrea Phillipou, Judith Piccone, Rebecca Pinkus, Bronwyn Raykos, Paul Rhodes, Elizabeth Rieger, Sarah-Catherine Rodan, Janice Russell, Haley Russell, Fiona Salter, Susan Sawyer, Beth Shelton, Urvashnee Singh, Sophie Smith, Evelyn Smith, Karen Spielman, Sarah Squire, Juliette Thomson, Stephen Touyz, Ranjani Utpala, Lenny Vartanian, Sabina Vatter, Andrew Wallis, Warren Ward, Sarah Wells, Eleanor Wertheim, Simon Wilksch, Michelle Williams

**Affiliations:** 1 InsideOut Institute for Eating Disorders, Level 2, Charles Perkins Centre (D17), Faculty of Medicine and Health, University of Sydney, Sydney, NSW 2006 Australia; 2 Lambert Initiative for Cannabinoid Therapeutics, University of Sydney, Sydney, Australia; 3 School of Psychology, Faculty of Science, University of Sydney, Sydney, Australia; 4 Brain and Mind Centre, University of Sydney, Sydney, Australia; 5 Healthcare Management Advisors, Melbourne, Australia; 6 Sydney Local Health District, New South Wales Health, Sydney, Australia

**Keywords:** Pharmacotherapy, Eating disorders, Anorexia nervosa, Bulimia nervosa, Binge eating disorder, Adjunctive therapy, Alternative therapy

## Abstract

**Background:**

The current review broadly summarises the evidence base for pharmacotherapies and adjunctive and alternative therapies in the treatment of eating disorders and disordered eating.

**Methods:**

This paper forms part of a Rapid Review series examining the evidence base in the field of eating disorders. This was conducted to inform the Australian National Eating Disorder Research and Translation Strategy 2021–2030. ScienceDirect, PubMed and Ovid/Medline were searched for included studies published between 2009 and 2021 in English. High-level evidence such as meta-analyses, large population studies and randomised control trials were prioritised, and grey literature excluded. Data from included studies relating to pharmacotherapy, and to adjunctive and alternative therapies in eating disorders, were synthesised and disseminated in the current review.

**Results:**

A total of 121 studies were identified, relating to pharmacotherapy (n = 90), adjunctive therapies (n = 21) and alternative therapies (n = 22). Some of the identified studies involved combinations of the above (e.g. adjunctive pharmacotherapy). Evidence of efficacy of interventions across all three categories was very limited with few relevant high quality clinical trials. There was a particular scarcity of evidence around effective treatments for anorexia nervosa (AN). With treatment of bulimia nervosa (BN), fluoxetine has exhibited some efficacy leading to regulatory approval in some countries. With binge eating disorder (BED), recent evidence supports the use of lisdexamfetamine. Neurostimulation interventions show some emerging efficacy in the treatment of AN, BN and BED but some, such as deep brain stimulation can be highly invasive.

**Conclusion:**

Despite widespread use of medications, this Rapid Review has identified a lack of effective medications and adjunctive and alternative therapies in the treatment of EDs. An intensification of high-quality clinical trial activity and drug discovery innovation are required to better assist patients suffering from EDs.

**Supplementary Information:**

The online version contains supplementary material available at 10.1186/s40337-023-00833-9.

## Abstract (plain language summary)

Eating disorders have the highest mortality rates and treatment costs of all mental health conditions. This rapid review summarises the evidence around the use of medications and various alternative therapies in the treatment of eating disorders. The review highlights a lack of effective interventions for the treatment of anorexia nervosa with an urgent need to trial new treatments for this condition. Two medications show some efficacy in treating other eating disorders: the antidepressant drug fluoxetine for the treatment of bulimia nervosa, and the stimulant drug lisdexamfetamine for binge eating disorder. There is some positive evidence emerging from novel therapies that involve brain stimulation technologies. Overall, more high-quality research is needed to discover and develop new medications, and other alternative therapies, to better assist patients with eating disorders.

**Supplementary Information:**

The online version contains supplementary material available at 10.1186/s40337-023-00833-9.

## Introduction

Eating disorders (EDs) are serious and potentially life-threatening mental illnesses characterised by persistently disrupted eating behaviours [1]. Frequently co-occurring with poor self-image, anxiety, and depressive disorders, EDs are associated with impaired physical health and psychosocial problems that diminish quality of life. The DSM-5 (American Psychiatric Association, 2013) [2] currently recognises three primary EDs: Anorexia Nervosa (AN), Bulimia Nervosa (BN) and Binge Eating Disorder (BED). The DSM-5 additionally recognises Other Specified Feeding or Eating Disorder (OSFED), Unspecified Feeding or Eating Disorder (UFED), Avoidant/Restrictive Food Intake Disorder (ARFID), Pica, and Rumination Disorder (RD). EDs have among the highest mortality rates of any mental illness and are considered one of the most difficult psychiatric conditions to manage and treat [3]. EDs are particularly treatment-resistant, with over 50% of ED patients reaching a severe and enduring stage of the illness [3].

**Fig. 1:**
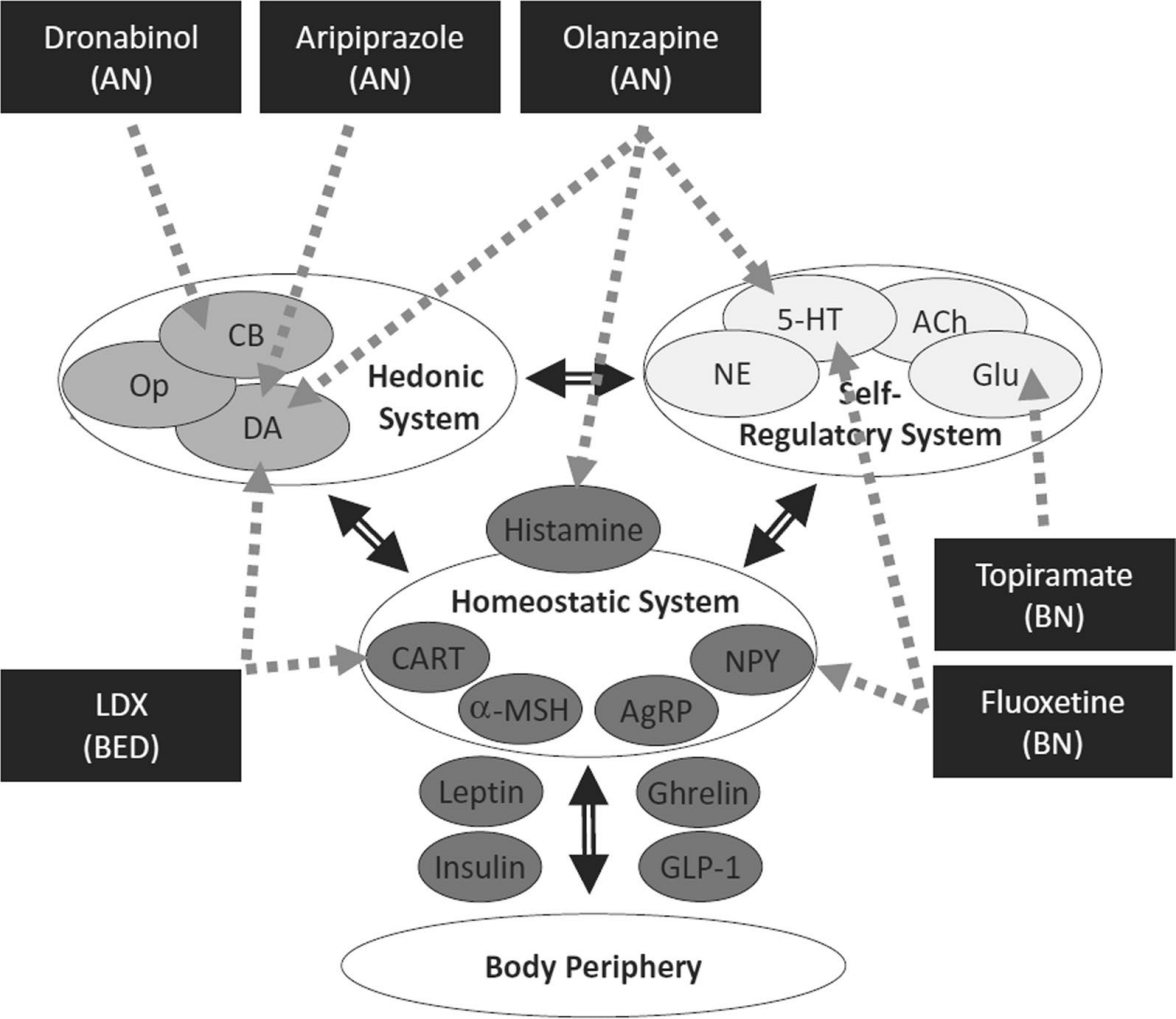
Schematic showing pharmacotherapies that have been investigated in the treatment of the three core EDs (AN, BN and BED) and their conceptual modes of action. This includes the hedonic (cannabinoid, opioid and dopamine-related), self-regulatory (serotonin and glutamate-related), homeostatic (histamine, CART and neuropeptide Y related) and peripheral metabolic (leptin, ghrelin, insulin GLP-1) systems. Source: Himmerich & Treasure (2018) ‘Psychopharmacological advances in eating disorders’

Despite substantial research into ED aetiology and pathology, few existing theoretical models have been translated into effective interventions [4]. The mechanisms underlying ED symptomatology are complex and multi-factorial and involve a wide range of potential therapeutic targets. Research has examined whether various pharmacotherapies and alternative therapies are effective in the treatment of EDs and how these interventions interact with specific neural and neuroendocrine circuits to influence relevant behaviours [5]. Pharmacological targets for interventions include the monoaminergic transporters (e.g., serotonin and norepinephrine) and receptors (e.g. dopamine) that are implicated in mood, attention, motivation and various self-regulatory processes. Other interventions target receptors implicated in the rewarding aspects of food-related stimuli such as opioids and endocannabinoids. Additional appetite-related neuroendocrine targets include hypothalamic and peripheral signalling molecules such as ghrelin, leptin, neuropeptide Y, and glucagon-like peptides [5]. Figure 1 summarises potential targets engaged by various pharmacotherapies.

The most commonly prescribed psychotropic medications in EDs are serotonergic antidepressants (e.g. fluoxetine) and atypical antipsychotics (e.g. olanzapine and aripiprazole), with some additional prescription of mood-stabilising medications and anxiolytics [6]. However, the only pharmacotherapies with a solid evidence base and widespread regulatory approval are the SSRI antidepressant fluoxetine for BN, and the stimulant lisdexamfetamine, for BED [5]. In AN, no pharmacotherapies are recommended as a first line treatment [6]. Considering the high rates of relapse and treatment attrition among individuals with ED, the lack of effective pharmacotherapies and alternative therapies is of significant concern [3, 7].

Innovative treatments involving novel neurostimulation technologies (including transcranial magnetic stimulation, transcranial direct current stimulation, neurosurgical ablation, deep brain stimulation) have shown some promise in the treatment of AN, BN and BED, although the invasive nature of some of these interventions may limit their practical use given legitimate safety concerns [8].

The current Rapid Review (RR) paper is one of a series scoping the field of eating disorders, commissioned by the Australian Federal Government to inform the Australian National Eating Disorders Research and Translation Strategy 2021–2031. This review aims to identify and broadly summarise the evidence around various pharmacotherapies and adjunctive and alternative therapies in the treatment of eating disorders and disordered eating.

Alternative therapies are emerging interventions that are yet to be considered as conventional standards of care and include interventions such as neuromodulation or exercise therapy. Adjunctive, or complimentary therapies, aim to facilitate the outcomes of the primary treatment, for example adjunctive pharmacotherapy with fluoxetine being added to a primary treatment involving psychotherapy for anorexia nervosa. Accordingly, some of the studies identified in the Rapid Review involved pharmacotherapies or alternative therapies being used adjunctively, in combination with other interventions. Overall, it is hoped that the current review may help inform health policy and clinical practice around EDs and stimulate future translational research.

## Methods

The Australian Government funded the InsideOut Institute for Eating Disorders (IOI) to develop the *Australian Eating Disorders Research and Translation Strategy 2021–2031* [9] under the *Psych Services for Hard to Reach Groups* initiative (ID 4-8MSSLE). The strategy was developed in partnership with state and national stakeholders including clinicians, service providers, researchers, and experts by lived experience (including consumers and families/carers). Developed through a two-year national consultation and collaboration process, the strategy provides the roadmap to establishing EDs as a national research priority and is the first disorder-specific strategy to be developed in consultation with the National Mental Health Commission. To inform the strategy, IOI commissioned Healthcare Management Advisors (HMA) to conduct a series of rapid reviews to broadly assess all available peer-reviewed literature on the six DSM-5 listed EDs.

A Rapid Review Protocol [10] was utilised to swiftly synthesise evidence in order to guide public policy and decision-making [11]. This approach has been adopted by several leading health organisations including the World Health Organisation [12] and the Canadian Agency for Drugs and Technologies in Health Rapid Response Service [13], to build a strong evidence base in a timely and accelerated manner, without compromising quality. A rapid review is not designed to be as comprehensive as a systematic review—it is purposive rather than exhaustive and provides actionable evidence to guide health policy [14].

The rapid review provides a narrative synthesis that adheres to the PRISMA guidelines [15]. It is divided by topic area and presented as a series of papers. Three research databases were searched: ScienceDirect, PubMed and Ovid/Medline. To establish a broad understanding of the progress made in the field of eating disorders, and to capture the largest evidence base from the past 12 years (originally 2009–2019 but expanded to include an additional 2 years), the eligibility criteria for included studies into the rapid review were kept broad. Therefore, included studies were published between 2009 and 2021, in English, and conducted within Western healthcare systems, or health systems comparable to Australia in terms of structure and resourcing. The initial search and review process was conducted by three reviewers between 5th December 2019 and 16th January 2020. A subsequent update was then undertaken for the years 2020–2021 and was conducted by reviewers at the end of May 2021.

The rapid review had a translational research focus with the objective of identifying evidence relevant to developing optimal care pathways. Searches therefore used a Population, Intervention, Comparison, Outcome (PICO) approach to identify literature relating to population impact, prevention and early intervention, treatment and long-term outcomes. Purposive sampling focused on high-level evidence studies such as: meta-analyses, systematic reviews, moderately sized randomised controlled studies (RCTs) (*n* > 50), moderately sized controlled-cohort studies (*n* > 50), and population studies (*n* > 500).

Notably, relevant studies involving patients with diagnoses of ARFID and UFED were sparse and this necessitated a less stringent eligibility criterion due to the paucity of published articles in this domain. As these diagnoses are newly captured in the DSM-5 (released in 2013, within the allocated search timeframe), the evidence base is only formative. Thus, smaller studies (n =  < 20) and narrative reviews were also considered and included. Grey literature, such as clinical or practice guidelines, protocol papers (without results), and Masters theses or dissertations, were excluded.

Full methodological details including eligibility criteria, search strategy and terms, consort diagram, and data analysis are published in a separate protocol paper. The overall rapid review had a very large scope and included a total of 1320 studies [16] (see Additional file 1 for PRISMA flow diagram). Studies relating to pharmacotherapies and adjunctive and alternative therapies represented less than 10% of the overall review and are synthesised and presented in the current review. No further analysis was carried out on reported results.

## Results

The rapid review identified 121 studies relating to pharmacotherapy (n = 90), adjunctive therapies (n = 21) and alternative therapies (n = 22). Some of the studies identified in the Rapid Review involving pharmacotherapies and alternative therapies were also adjunctive (i.e. trialled in combination with other interventions). Some studies identified in the Rapid Review used the “Eating Disorder Not Otherwise Specified” (EDNOS) category from the DSM-IV-TR. Since those papers were published, the DSM-5 replaced this category with “Other Specified Feeding or Eating Disorder” (OFSED) and “Unspecified Feeding Or Eating Disorder” (UFED). Given the scale of the overall rapid review, evidence on pharmacotherapy, adjunctive and alternative therapies made up only 9% of the studies identified, outlining a lack of research in this area of unmet need. A full list of included studies for this topic including population, aims and outcome measures can be found in Additional file 2: Table S1.

## Pharmacotherapy

### Anorexia Nervosa

There are currently no pharmacological interventions recommended for the treatment of AN [17]. Historically, tricyclic antidepressants (TCAs) and first-generation antipsychotics were examined, but results were not clinically promising and involved a notable side effect burden [6].

More recently, a variety of selective serotonin reuptake inhibitor (SSRI) antidepressants and second-generation antipsychotics (SGA) have been investigated, yielding mixed results overall. Despite marginal efficacy, SSRIs and SGAs are very commonly prescribed in clinical practice to treat AN [18, 19]. While these drugs have some demonstrated efficacy in BN and BED [18] (see below), there is only weak evidence demonstrating efficacy in promoting weight gain, reducing eating disorder symptoms, or preventing relapse in AN [20–23]. Arguments for their continued use rest on the assumption they may help effectively manage comorbidities such as anxiety or depressive disorders, act as helpful adjuncts to psychotherapy, assist with weight restoration, improve treatment retention or prevent relapse [6, 24–27]. However, there is sparse evidence in support of these assumptions [18].

#### Antidepressants

SSRIs [6, 24] and TCAs have very limited clinical benefits in individuals with AN. Due to the poor tolerability of TCAs and their lethality in overdose, they are not recommended as a treatment for AN today [5, 28]. No RCT has demonstrated significant clinical efficacy of SSRIs in the treatment of AN and no relevant RCTs have been published in the past 12 years. The RCTs identified by systematic reviews [6, 20] are typically very dated (1998–2006). Despite this, SSRIs continue to be widely prescribed off-label for AN.

##### Fluoxetine

Systematic reviews [6] identified by the Rapid Review reported two double blind RCTs [29, 30] investigating the SSRI fluoxetine following acute weight restoration in AN. Kaye et al. [29] reported that a small number of patients treated with fluoxetine (n = 16) had a significantly lower rate of relapse, an increase in weight and a reduction in eating disorder symptoms compared to those treated with placebo (n = 18). However, a larger RCT [30] in weight-restored patients (n = 93) found there was no difference in relapse or depressive symptoms in those treated with fluoxetine (n = 49) compared to placebo (n = 44) [30].

##### Citalopram

Flament et al. [6] identified one RCT [31] that investigated the SSRI citalopram. Weight gain was similar in both active drug and placebo groups, although citalopram appeared to improve depression, obsessive–compulsive symptoms, impulsiveness and trait-anger [6].

##### SSRIs in children and adolescents

The systematic review by Balestrieri et al. [20] identified one trial [32] that retrospectively compared 19 partially weight restored adolescents (n = 19) treated with the SSRIs fluoxetine, fluvoaxamine or sertraline to unmedicated adolescent patients (n = 13). They found no differences in BMI, eating disorder symptoms, obsessive–compulsive scores, or depression.

#### Antipsychotics

Second generation antipsychotics (SGAs), also known as atypical antipsychotics, have a complex pharmacology primarily involving dopamine and serotonin receptor antagonist (or partial agonist) effects. Weight gain and associated metabolic dysfunction is a common side effects of these agents when used in the treatment of psychosis and is thought to at least partly reflect the anti-histamine and 5-HT_2C_ antagonist properties of these drugs.

SGAs are the most widely researched category of pharmacotherapy in AN with the agent’s olanzapine and aripiprazole attracting the most clinical trials. Overall, mixed results have been obtained concerning the ability of these agents to reduce eating disorder psychopathology and promote weight gain in AN [6, 24, 25, 33–36]. Significant challenges have been noted by researchers in patients with AN around these drugs. Specifically, patients who have an egosyntonic relationship with their low body weight, wish to retain it, and thus resist taking antipsychotics because of their known association with weight gain [37]. Thus, positive results from clinical trials around weight gain and BMI with SGAs may not always translate into real-world clinical utility where non-adherence with these drugs is frequently an issue.

##### Olanzapine

A double-blind, placebo-controlled, 10-week flexible dose trial [35] in which patients with AN (n = 34) were randomly assigned to either olanzapine plus day hospital treatment (n = 16), or placebo plus day hospital treatment (n = 18), reported that olanzapine resulted in a greater rate of increase in weight, including earlier achievement of target body mass index, and a greater decrease in obsessive symptoms [35]. Two placebo-controlled-RCTs [38, 39] also reported olanzapine to be effective at increasing weight/BMI in patients with AN, although reductions in depressive or obsessive/compulsive symptoms were not observed in these trials [38, 39].

Norris et al. [40], in a pair-matched retrospective cohort study in adolescents, concluded that olanzapine was no more effective than treatment as usual (TAU) at increasing BMI. While the group receiving olanzapine gained weight faster and weighed more than the comparator group at discharge, the results were not statistically significant [40].

A systematic review by Marquez et al. [26] concluded that olanzapine did not result in any significant differences in weight gain when used as an adjunct to SSRIs. AN patients receiving olanzapine did, however, tend to stay in treatment longer and there was a significant advantage for olanzapine + SSRI compared to aripiprazole + SSRI, or SSRI alone, in reducing depressive symptomatology in participants with AN [41].

##### Aripiprazole

The Rapid Review identified a retrospective study of 106 adolescents with AN [36] where 22 individuals had been treated with the dopamine partial agonist antipsychotic drug aripiprazole. Drug treatment was associated with a greater increase in BMI compared to the control group that did not receive aripiprazole [36].

##### Quetiapine

A placebo-controlled trial [37] failed to demonstrate efficacy of quetiapine, another popular SGA, for weight gain in AN. However, a three-site trial comparing quetiapine to TAU [42] found weight gain was significantly greater in the active group compared to the TAU group [37, 42]. Active participants in this study also displayed a significant reduction in depressive symptoms [42]. There were important differences between the two sample populations and the designs involved in these studies with the Powers et al. [37] sample significantly older (mean age of 34) compared with Court et al. [42] and likely having a longer duration of illness [37, 42]. Longer duration of illness in AN is often linked with poorer outcomes and resistance to treatment, potentially contributing to the non-significant effect observed in the study sample [37, 43].

##### Risperidone

In a 12-week RCT trial involving the SGA Risperidone, no significant differences were found between the drug (n = 18) and placebo group (n = 22) in terms of weight gain [44], although a significant reduction in ‘drive for thinness’, a core feature of AN psychopathology, was observed in the earlier stages of the trial. However, this was not sustained through to trial completion [44]. This trial therefore failed to demonstrate a benefit of risperidone in adolescents with AN.

#### Cannabinoid receptor agonist

The endocannabinoid system, particularly CB_1_ cannabinoid receptors and their endogenous ligands anandamide and 2-AG, play an important role in modulation of both homeostatic and hedonic elements of appetite and food intake. Efforts to restore weight in AN patients have led to investigation of the potential benefits of cannabinoids, specifically Δ^9^-tetrahydrocannabinol (THC), predicated on the idea that these drugs would work to stimulate appetite and perhaps improve mood in affected individuals [45].

##### Dronabinol

The Rapid Review identified two RCTs investigating a cannabinoid receptor agonist, Dronabinol, which is a capsule containing THC. Both trials were conducted in groups of women with severe and enduring AN (of greater than five years illness duration). Andries et al. [45] reported that four weeks of daily Dronabinol was significantly more effective at promoting weight gain, with the active group gaining on average 0.73 kg more than the placebo group, without significant adverse events [45]. The second RCT assessed the effect of Dronabinol on physical activity in women with severe and enduring AN. Excessive exercise is a common symptom of AN and has been associated with greater illness severity in individuals with both AN and Purging Disorder (PD) [46]. While researchers hypothesised that low doses of the cannabinoid agonist would decrease physical activity in participants, increases in physical activity intensity were observed relative to controls, with no significant differences between groups in the amount of daily physical activity [47]. Systematic reviews [48, 49] identified by the current Rapid Review reference the aforementioned studies.

#### Oxytocin

Oxytocin is a neuropeptide with a central role in the regulation of appetite, feeding-related behaviours, social cognition, fear and anxiety [50]. Administration of oxytocin can modulate learning, attention, memory, social skills, trust, empathy and repetitive behaviours [51]. Some studies have achieved promising results with oxytocin in the treatment of obsessive compulsive disorder, social phobia and autism, conditions that are frequently co-morbid with AN and that share core neuropsychiatric features [51]. These include poor set shifting, cognitive rigidity, social anxiety, perfectionism, and obsessional behaviours. Thus, oxytocin has been identified as a neuropeptide of interest worth exploring in the treatment of AN with predictions that it may modulate attentional processes away from food- and body-related stimuli [50]. Moreover, lowered concentrations of oxytocin have sometimes been measured in patients with AN [51].

The Rapid Review identified 4 relevant RCTs investigating effects of intranasally delivered oxytocin in AN. Kim et al. [50] conducted a double-blind placebo-controlled within-subjects crossover study in 64 participants, 31 with AN and 33 healthy controls. The AN group showed significant reductions in the attentional biases towards eating-related stimuli and toward negative shape stimuli under the influence of 40 International Units (IU) of intranasal oxytocin (IN-OT). However, this was not associated with an increase in calorie consumption [50]. In a follow-up publication, Kim et al. [52] examined the impact of 40 IU IN-OT on consummatory behaviour and emotional recognition in AN patients relative to healthy controls. They found no effect of IN-OT on consummatory behaviour or on emotional recognition sensitivity [52].

Leppanen et al. [53] reported that a single dose of intranasal oxytocin (IN-OT) did not increase calorie consumption in individuals with AN over placebo [50, 52, 53]. Lowered concentrations of salivary cortisol measured in response to a laboratory food challenge indicated that oxytocin may reduce the heightened stress and fear of mealtimes in individuals with AN [53]. This was partially supported by a subsequent placebo-controlled RCT [54] conducted in women with AN who self-administered IN-OT daily for 4–6 weeks. After 4 weeks, salivary cortisol concentrations were significantly lowered in anticipation of an afternoon snack compared to placebo, as well as reduced baseline concentrations of salivary cortisol (post-treatment) [54]. However, morning plasma OT concentrations did not change after chronic IN-OT or with weight restoration. Lower salivary cortisol concentrations in response to IN-OT might suggest a reduced neuroendocrine stress response to food and eating. Researchers also concluded that IN-OT might enhance nutritional rehabilitation in AN by reducing eating concern and cognitive rigidity, although no significant impact on BMI change was shown. Overall effects of oxytocin in AN therefore appear to be subtle and most likely clinically insignificant.

#### Oestrogen

Concentrations of the gonadal steroid oestrogen are often low in individuals with AN [55]. Studies indicate that oestrogen impacts eating behaviour, cognitive function, body shape perception, mood and anxiety [55]. Further, oestrogen is commonly provided as an adjunctive therapy to females with AN to treat bone loss [55]. The Rapid Review identified a few trials that examined the effect of oestrogen replacement therapy on appetite regulation and neuropsychological deficits in AN [56]. Misra et al. [55] reported that oestrogen replacement therapy in adolescent females with AN was not superior to placebo groups in reducing ED symptoms or body shape concern but did provide a significant reduction in trait anxiety in the study population. This study also outlined that participants with restored oestrogen levels were less stressed by weight gain [55].

Preliminary evidence shows that high doses of oestrogen may benefit patients with Major Depressive Disorder (MDD), which is often comorbid with AN [3, 57]. It has also been proposed that oestrogen may increase the efficacy of SSRI treatment in MDD [57], which may be of benefit given that SSRIs appear to have very limited efficacy in treating AN [17].

#### Ghrelin and relamorelin

Ghrelin is a hormone produced by the enteroendocrine cells of the gastrointestinal tract that has been extensively studied as an appetite stimulating hormone [58]. Ghrelin decreases gastric emptying times which is sometimes associated with appetite stimulation and weight gain. The synthetic ghrelin analogue, relamorelin, has similar appetite stimulatory effects and was studied in a small parallel-design RCT involving 22 outpatient women with AN. Relamorelin was found to significantly reduce gastric emptying times and increase weight gain after 4 weeks of treatment (n = 10) compared to the group receiving placebo (n = 12) [58]. While larger RCTs and replication are clearly required, these early results suggest some potential in this novel therapeutic approach in AN.

#### Adipokines

Adipokines are signalling proteins released by fat cells (i.e., adipocytes). Recent evidence suggests that adipocyte signalling, particularly the release of vaspin, leptin, adiponectin, resistin, omentin and visfatin, may be dysregulated in AN and that this dysregulation may underpin weight loss in patients [59]. Potential targets within the gastrointestinal and associated immunological receptor pathways may represent an opportunity for efficacious pharmacotherapies for AN in future research [59]. However, human studies are yet to be undertaken [5].

#### Nutrient supplementation

Current research in nutrient supplementation has mainly been demonstrated in animal models or in trials with very small sample sizes [60, 61]. Research focused on the gut-brain axis has examined nutrient supplementation with probiotics, essential fatty acids and amino acids such as glutamine and tryptophan [60, 61].

##### Essential fatty acids

Supplementation with essential fatty acids can improve appetite and restore weight in a number of health conditions [62]. Researchers propose that the use of personalised fatty-acids as an adjunctive therapy in AN may reduce food aversion and food-based anxiety in patients. However, there is currently little evidence to support this theory [62]. A double-blind, placebo-controlled randomised trial in 24 adolescents with AN failed to demonstrate an advantage of 12 weeks of omega-3 polyunsaturated fatty acid supplementation (n = 12) over placebo (n = 12) in reducing ED and depressive symptoms or trait anxiety [63].

##### Tryptophan

Food restriction and excessive dieting can reduce circulating tryptophan, the amino acid precursor of serotonin, in individuals with AN. It has been hypothesised that that deficits in tryptophan may reduce the efficacy of SSRIs and SGAs [25] and therefore supplementation with tryptophan may be worthwhile [60]. However, one small RCT [64] found no advantage of fluoxetine plus nutritional supplements (tryptophan and essential fatty acids) over fluoxetine plus placebo.

### Bulimia nervosa

A relatively small evidence-base was identified involving pharmacotherapies for BN. As with AN, many of the RCTs identified were notably dated. Although the SSRI fluoxetine is an approved treatment for BN by the Food and Drug Administration (FDA) and the Therapeutic Goods Administration (TGA), few recent studies were identified by the Rapid Review showing effectiveness in this population. Significant improvements in binge and purge symptoms have been reported in some historical studies with the anticonvulsant and anti-craving drug topiramate. However, these RCTs are also significantly dated with no recent RCTs conducted.

#### SSRI antidepressants

A systematic review of RCTs [65] concluded that fluoxetine reduces binge eating episodes in individuals with BN by around 50–67%, reduces purging by around 50–56%, and may also prevent relapse [65]. Most RCTs were published in the 1990s and early 2000s. Another systematic review [66] of RCTs concluded, largely on the basis of studies from the 1990s, that fluoxetine and citalopram significantly reduce binge-eating episodes and purging symptoms [66].

A more recent large RCT [67] examined early response to fluoxetine in a large cohort of BN patients (*n* = 785). Findings indicated that a favourable response to fluoxetine could be predicted by week three and that patients who achieved less than a 60% reduction in binge eating frequency after three weeks of treatment should be considered non-responders and be allocated to a different treatment modality. This represents useful clinical guidance for the clinical management of patients with BN [67].

#### Anticonvulsants

##### Topiramate

The anticonvulsant drug topiramate, is used in the treatment of generalised and focal seizures in epilepsy and in alcohol dependence. It has a complex mode of action influencing sodium and calcium ion channels and influencing glutamate and GABA activity. As identified in the systematic review by Hay and Claudino [66] there has been one RCT [68, 69] examining the efficacy of topiramate on binge purge frequency and associated psychiatric outcomes in BN. BN patients were randomised to topiramate (n = 35) or placebo (n = 34) for 10 weeks. Topiramate was initially administered at 25 mg/day and then titrated up by 25–50 mg/week to a maximum of 400 mg per day over 6–8 weeks. Topiramate treatment significantly improved binge and purge symptoms with associated improvements in eating behaviours, anxiety, self-esteem and body image at 10 weeks compared to placebo. Adverse events reported included fatigue, influenza-like symptoms, paresthesia, hypoesthesia, nausea, constipation, difficulty with concentration/attention, headache and nervousness. No serious adverse events were reported [68].

#### CNS stimulants

##### Lisdexamfetamine

As described below, the stimulant drug lisdexamfetamine (LDX) is now a first line treatment for Binge Eating Disorder (BED) and has been explored in BN. A small open-label 8-week study [70] examined feasibility, efficacy and safety of LDX in 23 adults with BN. Reductions in binge eating episodes and compensatory behaviours were observed relative to baseline in the 18 participants completing the trial [70]. Although results were encouraging, the sample size was small and future large RCTs are clearly needed to confirm the efficacy and safety of LDX in BN [70].

#### Oxytocin

A double-blind single dose within-subject crossover trial reported that a single dose of intranasal oxytocin (40 IU) reduced calorie consumption over 24 h compared to healthy controls. Investigators also reported that oxytocin increased emotional recognition sensitivity in participants with BN (n = 34) and healthy controls (n = 33) [52]. Further research is required to confirm the clinical utility of oxytocin in the treatment of BN.

#### Antibiotics

##### Erythromycin

Altered gastric emptying and abnormal postprandial release of hormones that regulate satiation may contribute to binge eating behaviours in BN due to a reduction in satiety cues [71]. The antibiotic erythromycin is a prokinetic agent, an agonist of motilin receptors, which stimulates gastric motility. A randomised double-blind treatment with erythromycin compared rate of gastric emptying and postprandial hormone release in women with BN (n = 32) compared to controls (n = 24). Erythromycin and placebo were then randomised in the patients with BN. Those randomised to erythromycin had increased gastric emptying. However, there were no differences in postprandial release of hormones or any difference in clinical response [71].

### Binge eating disorder

A relatively large body of evidence was identified around the use of pharmacotherapies in BED. A significant proportion of studies related to CNS stimulants used for ADHD treatment, particularly lisdexamfetamine (LDX) [72, 73] which has been consistently shown to reduce the frequency of binge eating. Other ADHD medications such as methylphenidate and dasotraline, have also demonstrated some efficacy at reducing binge eating episodes. Studies on other pharmacotherapies including SSRIs, anticonvulsants, norepinephrine/dopamine reuptake inhibitors, and anti-obesity medications are less conclusive [73, 74]. There is some evidence of efficacy of tricyclic antidepressants (TCA) in BED, however, they are not recommended due to significant adverse effects [6].

#### Lisdexamfetamine

LDX is an inactive prodrug that is converted by the body to d-amphetamine, a CNS stimulant that promotes the release and inhibits the reuptake of dopamine and norepinephrine. LDX is commonly used in the treatment of ADHD, and is the only treatment for BED that is currently approved by the Food and Drug Administration (FDA) and the Therapeutic Goods Administration (TGA) [75]. LDX, like all amphetamine stimulants, has direct appetite suppressant effects that may be therapeutically useful in BED, although long-term neuroadaptations in dopaminergic and noradrenergic systems caused by LDX may also be relevant, leading to improved regulation of eating behaviours, attentional processes and goal-directed behaviours [76].

In one study examining neural activation in response to palatable food, individuals with BED exhibit greater brain activation in five regions of interest (ventromedial prefrontal cortex, ventrolateral prefrontal context, striatum, globus pallidus, thalamus), compared to a control group of obese women without BED [77]. LDX reduces this response, decreasing binge eating frequency and obsessive/compulsive symptoms [77]. A meta-analysis reported that LDX was approximately 1.7 times more effective at reducing obsessive/compulsive symptoms in individuals with BED than antidepressants [78].

Since 2013, the evidence-base supporting effectiveness of LDX has grown significantly, consolidating LDX as a first-line treatment for BED [79]. LDX approval for the treatment of BED was reliant on a clinical program carried out by a single research group. The program included one Phase II proof-of-concept, placebo controlled study, testing fixed doses of LDX 30, 50 and 70 mg/day [80], two phase III dose optimisation 12-week placebo-controlled studies examining LDX 50-70 mg/day [81, 82], one withdrawal study and one long-term safety study [83, 84].

McElroy et al. [80] randomised participants (1:1:1:1) to receive placebo or 30, 50, or 70 mg/day of LDX. Assessment of dose response found that at least 50 mg/day and 70 mg/day doses were required to be effective at reducing binge eating frequency and ED symptomatology and were associated with higher rates of abstinence compared with placebo [80]. These results were further confirmed by a review which demonstrated statistical and clinical superiority of 50 mg/day or 70 mg/day of LDX compared to placebo [85].

The two 12-week phase III identically randomised, placebo-controlled, parallel group, multicentre short-term trials in adults with moderate to severe BED (n = 374 and n = 350) were performed across the US, Germany, Sweden, Spain and Canada [81, 82]. Participants were randomised 1:1:1 to receive 12 weeks of dose-optimised LDX (50 or 70 mg) or placebo. In the LDX group, 36.2% and 40% of participants achieved four-week cessation from bingeing at the end of the 12-week treatment period compared to 13.1% and 14.1% in the placebo group. Post-hoc analysis by gender and age further showed the drug to be effective at reducing binge frequency across genders and age subgroups within the population sample [86]. A meta-analysis of the Phase II [80] and Phase III [81, 82] trials reported four-week binge cessation rates to be 39.6% with LDX compared to 14.7% with placebo [87].

A multinational, double-blind, placebo-controlled, randomised withdrawal study assessed maintenance of efficacy of LDX and risk of binge eating relapse in adults with moderate to severe BED [84]. Following a 12-week, open-label phase II trial, 418 participants with moderate to severe BED received dose optimised LDX (50 or 70 mg/day) [84]. A total of 275 LDX responders were identified (defined as ≤ 1 binge eating day per week for 4 consecutive weeks) and this cohort were further randomised 1:1 to placebo (n = 138) or continued LDX (n = 137) during a 26-week, double-blind, randomised withdrawal phase [84]. Risk of binge-eating relapse over 6 months was lower in participants continuing LDX than in those randomised to placebo. The observed proportions of participants meeting relapse criteria were 3.7% (5 of 136) for LDX and 32.1% (42 of 131) for placebo [84]. Risk of relapse was estimated to be 11 times lower in the LDX group than the placebo group [84].

LDX is considered to be relatively safe, although notable side effects include irritability, jitteriness, dry mouth, headache, decreased appetite, weight loss, respiratory tract infection, and insomnia [76, 80, 81, 85]. A 12-month, open-label study assessing long-term safety and tolerability of LDX in adults with BED reported relatively low rates of adverse events [83]. Only a small proportion (9%) of participants discontinued treatment due to treatment-emergent adverse events (TEAE) or reported severe or serious side-effects. TEAEs reported in greater than or equal than 10% were dry mouth, headache, insomnia, and upper respiratory tract infection. All TEAEs had resolved at the end of the 12-month period [83].

One trial identified by the RR, did not report statistically significant differences between LDX and placebo groups for reduction in binge eating frequency however reported significantly greater weight loss and reduction in BMI [76]. This was a single-centre, 12-week, double-blind, parallel-group and flexible dose study that randomised 50 participants with moderate to severe BED to dose optimised (50 or 70 mg/day) LDX (n = 25) or placebo (n = 25) [76].

Finally, the Rapid Review identified a study assessing the cost-effectiveness of LDX versus no pharmacotherapy in adults with BED assuming a 12-week course of treatment. While the cost to the patient was higher compared with no pharmacotherapy, patients on LDX gained an increase in quality of life which justified the willingness to pay for this pharmacotherapy [88].

Evidently, there is a substantial volume of trials with high-quality evidence supporting the efficacy of LDX in reducing binge eating frequency in treatment of adults with moderate to severe BED at 50–70 mg/day.

#### Methylphenidate

Methylphenidate is another CNS stimulant commonly used in the treatment of ADHD, exerting its effect through a selective inhibition of dopamine reuptake. Methylphenidate is widely available in generic form meaning it is more affordable than LDX. A 12-week study randomised 49 women with BED to methylphenidate (n = 22) or Cognitive Behavioural Therapy (CBT) (n = 27) [89]. The active drug group were on a flexible-dosing range from 18 to 72 mg/day. Results showed that while both treatments were effective at reducing binge eating frequency, in patients with BED, methylphenidate caused a greater reduction in BMI compared to CBT alone [89].

Davis et al. [90] examined sex differences in response to a single dose of methylphenidate, and whether these responses were moderated by BED status. This was a double-blind, crossover design comparing adults with BED (n = 90) to those without BED (n = 108) [90]. Significant reductions in appetite, food craving and consumption compared to placebo were only reported in females [90]. They also found that BED status did not moderate any of the drug-placebo differences. Researchers suggest the possibility that CNS stimulants may be less effective in men in an acute paradigm, although these findings are inconsistent with the aforementioned study [86] reporting efficacy of chronic LDX at reducing binge eating frequency across genders. Overall, these results provide additional support for the efficacy of CNS stimulants in reducing BED symptomatology.

#### Armodafinil

Armodafinil is a wakefulness-promoting agent used in the treatment of excessive sleepiness caused by narcolepsy and sleep apnoea. The primary mode of action is antagonism of orexin receptors which are associated with both wakefulness and appetite and there are also secondary actions involving dopamine receptor agonist effects which may help regulate dopamine dysfunction in BED. A small placebo controlled 10-week RCT [91] studied the efficacy of armodafinil (150–250 mg/day) in patients with BED. The results of the trial were inconclusive. The initial analysis did not reveal significant differences in reducing binge eating frequency between the active (n = 30) and placebo (n = 30) groups. However the investigators reported that armodafinil was associated with a greater reduction in binge eating frequency and obsessive/compulsive symptoms compared to the placebo group [91].

#### Antidepressants

##### Dasotraline

This drug is a serotonin-norepinephrine-dopamine reuptake inhibitor (SNDRI) initially developed for ADHD treatment. The Rapid Review identified two placebo-controlled double-blind studies of dasotraline [92, 93]. One RCT involved a sample of 486 patients randomised (1:1:1) to receive 12 weeks of once-daily fixed doses of dasotraline (4 or 6 mg), or placebo [92]. At week 12, treatment with 4 mg did not have any significant results, while active treatment with 6 mg/day significantly reduced binge eating days per week and obsessive/compulsive symptoms compared to placebo [92].

The second RCT randomised 315 patients with BED to receive 12 weeks of once-daily flexible doses (4, 6, or 8 mg/day) of dasotraline or placebo [93]. Patients randomised to dasotraline were initially treated with 4 mg/day then titrated to 6 mg/day by week 4. The dose of dasotraline could then be adjusted in increments or decrements of 2 mg/day depending on efficacy and tolerability for each patient [93]. Treatment with dasotraline was associated with a significantly greater reduction in binge eating per week at end of treatment. Treatment with dasotraline was associated with greater mean reduction in weight and BMI compared to placebo at week 12, with improvements notable as early as week 1 [93]. The most frequent adverse events reported were insomnia, dry mouth, decreased appetite, anxiety, nausea, decreased weight and headache [93].

##### Vortioxetine

This drug is a newer style of antidepressant that exerts its effects via enhancing levels of serotonin, noradrenaline, dopamine and acetylcholine. A double-blind, parallel design, placebo-controlled study trial identified by the Rapid Review examined the efficacy of vortioxetine in BED. [94]. Eighty adults with BED received a 12-week treatment of vortioxetine (10 mg/day for one week then, then titrating up to 20 mg/day) or placebo. Vortioxetine treatment was no more effective than placebo at inhibiting binge eating or reducing BMI [94].

##### Bupropion

This drug is a norepinephrine-dopamine reuptake inhibitor antidepressant that reduces cravings for nicotine and has been widely used in smoking cessation treatment. Bupropion also reduces food cravings and has shown to significantly reduce weight in obese patients. One RCT involved a sample of 61 overweight women with BED who were randomised to receive bupropion (300 mg/day) or placebo for 8 weeks. Investigators reported a modest weight reduction among participants in the active group compared with placebo [95]. However, bupropion did not improve binge eating, food craving or depressive symptoms compared to placebo [95].

#### Opioid receptor antagonists

Opioid receptors, particularly of the µ subtype, influence appetite control and hedonic processing associated with food-related stimuli. Opioid receptor antagonists such as naloxone and naltrexone generally suppress appetite for palatable food in animal models and there is some preliminary support for their use in the treatment of BED.

##### GSK1521498

One double-blind parallel design, fixed dose study trialled the effects off the selective μ opioid receptor antagonist GSK1521498 on attentional bias to food cues, working memory, sustained attention, and psychomotor speed in 63 severely obese adults with BED. Participants were given a 1-week placebo run-in, then randomised to 4 weeks of 2 mg/day or 5 mg/day of GSK1521498 or placebo [96]. Results demonstrated that 5 mg/day GSK1521498 significantly reduced attentional bias to food cues [96] without affecting working memory, sustained attention, or psychomotor speed.

##### ALKS-33

A placebo-controlled pilot study did not find any benefit of treatment with ALKS-33 (a μ opioid receptor antagonist also known as samidorphan) compared to placebo for binge eating frequency, obsessive/compulsive symptoms, ED symptoms or any other BED related measures in women with BED and obesity [97].

##### Naltrexone extended release + bupropion extended release (NB)

The combination of the μ opioid receptor antagonist, naltrexone, with the antidepressant bupropion (NB) is approved in the US as an anti-obesity agent. A 24-week open-label, single arm trial investigated NB combination therapy (32 mg naltrexone ER + 360 mg bupropion ER) in 25 obese women with MDD and suspected BED. NB treatment was associated with a significant reduction in binge eating symptoms, weight loss and depressive symptoms [98].

A small placebo-controlled double blind RCT evaluated the effects of NB in 22 adults with BED with comorbid obesity. Participants were randomised to receive 12 weeks of NB (naltrexone 50 mg + bupropion 300 mg) or placebo. Results were inconclusive. In the group that received NB there was a reduction in binge eating, eating disorder psychopathology, depression and weight when compared to baseline measurements. Outcomes remained improved at 6-month follow-up following discontinuation of medication. However, results were not superior to the placebo group [99]. Larger scale RCTs are required to better determine the efficacy of NB in BED [99].

#### GABA and glutamate acting drugs

Some research indicates that the anticonvulsant drugs acamprosate and topiramate, both used in treating addictions as well as in epilepsy, may have potential in the treatment of BED. Both provide a subtle modulation and rebalancing of gamma amino-butyric acid (GABA) and glutamate systems which play an important role in regulation of food intake as well as the craving for alcohol and other drugs of abuse [100].

##### Acamprosate

Acamprosate is a glutamate receptor modulator which is approved in the treatment of alcohol dependence and has been reported to reduce food craving and weight in individuals with alcohol dependence [100]. A 10-week, randomised 1:1, placebo-controlled, flexible dose trial tested acamprosate in 40 patients with BED [100]. Dosing commenced at 1998 mg/day for the first two weeks and was then titrated up to 2997 mg/day until week 10. Results were inconclusive. Investigators reported that acamprosate was not superior to the placebo in reducing binge eating frequency, body weight, or BMI. However, secondary analysis showed significant decreases in binge days per week, obsessive/compulsive symptoms, and weight loss in the active group when compared to baseline measurements [100].

##### Topiramate

The anticonvulsant drug topiramate, discussed above in relation to BN, has also been successfully used in the treatment of BED [101]. A systematic review and meta-analysis of three RCTs involving topiramate vs placebo involving a total of 528 BED patients concluded that topiramate was significantly more effective than placebo at reducing binge episodes and producing body weight reduction [101]. However, the RCTs were significantly dated (2003–2007) and the participants treated with topiramate withdrew at higher rates due to adverse events, compared to placebo. Adverse events reported included paresthesia, taste perversion, confusion, upper respiratory tract infection, leg pain, memory, and concentration difficulties. Serious adverse events were reported in one RCT including acute cholecystitis, tibial fracture, and major depression [101].

#### Chromium picolinate

Metabolic dysregulation in individuals with BED has prompted investigation into agents which can help modulate glycaemia to reduce increased risk of developing abnormal glucose metabolism [102]. Chromium picolinate (CrPic) is a nutritional supplement that is commonly used to treat type 2 diabetes and in weight loss. One double-blind placebo-controlled trial in 24 individuals with BED were randomised to CrPic 1000 mcg/day, CrPic 600 mcg/day or placebo over a six-month period [102, 103]. Individuals randomised to a moderate (600 mcg/day) dose of CrPic had significantly reduced fasting glucose levels compared to placebo [103]. Interestingly, the moderate dose of chromium picolinate significantly improved insulin regulation relative to both the placebo and high dose groups (1000 mcg/day) [102].

#### Anti-obesity drugs

Studies identified by the Rapid Review indicate that a variety of anti-obesity drugs may be useful in the treatment of BED. Obviously, any drug that is effective at reducing weight in individuals with obesity might be expected to also be effective in reducing binge eating in patients with BED. Notably, around 7.5–30% of individuals with obesity seeking treatment have co-morbid binge eating disorder (BED) or subclinical binge eating disorder [104].

##### Rimonabant

A dysregulation in the endocannabinoid system contributes to the pathophysiology of obesity. The cannabinoid receptor 1 (CB1) antagonist, rimonabant has demonstrated efficacy in treating obesity albeit with the risk of significant neuropsychiatric side effects. A multicentre, randomised, double-blind, placebo-controlled study in 289 obese participants with BED found the group treated with rimonabant had significantly reduced body weight relative to placebo [105].

##### Liraglutide

Glucagon-like peptide-1 (GLP-1) is a hormone secreted from the small intestine in response to food, inhibiting appetite and delaying gastric emptying. Its satiating effects are therefore of interest in the treatment of BED. Liraglutide, a GLP-1 receptor agonist, has been approved for obesity. In one study, 44 participants with obesity and subclinical binge eating were randomly assigned to liraglutide or placebo. Participants randomised to liraglutide showed significant improvements in binge eating, and a reduction in body weight and BMI [104].

### Night eating syndrome

Night Eating Syndrome (NES) is the only other-specified ED (OSFED) for which evidence on pharmacotherapy has been identified [106].

#### Escitalopram

A 12 week RCT randomised 40 patients with NES to SSRI escitalopram (n = 20) or placebo (n = 20) [107]. The study found that although escitalopram was not effective at reducing night-time calorie consumption and NES scores, the active group had a slight decrease in weight than the placebo group [107]. Conversely, an open label trial trialled escitalopram for 12 weeks in 31 adults with NES [108]. Investigators reported significant reductions in body weight, night-time caloric intake and awakenings [108].

#### Sertraline

A review by Allison and Tarves [109] identified two open label trials and one double blind RCT trial of sertraline in NES. All studies reported significantly reduced night-time eating, awakenings, decreased caloric intake after the evening meal, NES scores and weight loss. However, these studies were significantly dated (2004–2006). No recent studies have been conducted [109].

#### Ramelteon

Ramelteon is a melatonin receptor agonist, which is used in the treatment of insomnia. One retrospective study investigated ramelteon in 49 adults with sleep related eating disorders and/or NES and subsequent dose reduction in benzodiazepine derivatives and Z-drugs (zopiclone/zolpidem) [110]. Mean eating frequency decreased per week and significant reductions in benzodiazepine and Z-drugs were reported from baseline to post-ramelteon treatment. These results indicate ramelteon may be efficacious in the treatment of sleep relating eating disorder, NES and subsequent dose reduction of benzodiazepines and Z-drugs.

### ARFID

Evidence regarding use of pharmacotherapy for cases of avoidant/restrictive food intake disorder (ARFID) are limited. Trials on the use of olanzapine, mirtazapine and buspirone as an adjunct to psychological interventions to treat children and adolescents with ARFID have typically been case reports or series, which limits their generalisability. Some evidence on increasing appetite and reducing anxiety has been reported however randomised controlled trials are needed to confirm their clinical utility in this population [21].

### OFSED, UFED, pica and rumination disorder

No research relating to pharmacotherapy interventions for OSFED (purging disorder (PD), atypical-AN (A-AN), subthreshold-BN (S-BN) and subthreshold-BED (S-BED)), UFED, Pica, or Rumination Disorder (RD) was identified.

## Combination and adjunctive therapy

Medications for eating disorders are commonly prescribed within a context of “treatment as usual” which may include psychotherapy, nutritional counselling, and support groups. As such many of the clinical trials of such medication referred to above incorporate medications within a broader therapeutic context. This is particularly the case with AN, where no medication is recommended as a primary treatment. Some studies, however, have formally examined the adjunctive role of medications and other psychological therapies. The distinct objective of evaluating the literature on combined therapies is to understand whether therapies provided in tandem is more effective than monotherapy.

Few studies have been identified that investigate the comparative effectiveness of combining psychotherapies and pharmacotherapies, with most studies conducted in BED and BN [111]. One study investigated CBT, nutritional support, and pharmacotherapy in 74 patients with different eating disorders, including AN (n = 32), BN (n = 19) and EDNOS (n = 23) [112]. Results indicated that all participants achieved similar remission rates as a result of their combined CBT, nutritional support, and pharmacotherapy treatments, however no information was found on types of pharmacotherapies provided to participants. In addition, there was no control group, making it difficult to assess the true efficacy of this combination of treatments [112].

### Binge eating disorder

A systematic review has examined 11 RCTs assessing the efficacy of combined psychotherapy and pharmacotherapy (antidepressants, anti-obesity medication, and antiepileptics) in the treatment of BED [113]. Findings from this review indicated that there was an advantage of combining CBT with the anticonvulsant drugs zonisamide and topiramate compared to CBT alone. On the other hand, there was no advantage in combining CBT or Behavioural Weight Loss Therapy (BWLT) with antidepressants and moderate advantage in combining CBT with anti-obesity drugs, compared to either CBT alone or CBT + placebo [113].

#### CBT & anticonvulsants

Two RCT’s have investigated anticonvulsants in combination with CBT for the treatment of BED. One RCT assessed CBT in combination with zonisamide which was shown to be more effective than CBT alone for reducing binge eating frequency. Over 24 weeks, CBT + zonisamide gave significantly greater reductions in eating disorder psychopathology, binge eating episodes, and weight loss compared to CBT alone. These results were maintained at 12-month follow-up in those receiving CBT + zonisamide [111]. A 21-week multi-centre, double-blind, parallel-group, randomized controlled trial in 73 patients with BED tested the anticonvulsant topiramate with CBT compared to CBT + placebo [113]. At 21 weeks topiramate added to CBT significantly improved depressive symptomology, binge eating scores and weight loss compared to CBT alone.

#### CBT & fluoxetine

The efficacy of CBT in combination with the SSRI fluoxetine was studied in a clinical trial with BED patients. The 12-month remission rates were 3.7% for the fluoxetine-only group, 26.9% for the CBT + fluoxetine group, and 35.7% for CBT + placebo [114]. This indicates that CBT is an effective treatment for reducing binge eating, but that the addition of an SSRI adds little to treatment outcomes [114]. Patients displaying higher levels of ‘overvaluation of shape’ were significantly less likely to achieve remission if receiving medication only.

#### CBT & Orlistat

In one study, 50 patients with BED received either guided self-help CBT (self-administered CBT guided by a therapist [shCBT]), combined with the anti-obesity medication orlistat or shCBT and placebo [115]. Combination therapy was more efficacious than the placebo condition, resulting in 64% of the active group achieving binge eating abstinence compared with 36% of the placebo group [115].

#### CBT + Sibutramine

Sibutramine is a norepinephrine, serotonin and dopamine reuptake inhibitor with strong appetite suppressant effects. One RCT randomised 104 obese patients with BED to sibutramine, placebo, guided self-help CBT (shCBT) + placebo, or shCBT + sibutramine [116]. Greater decreases in binge eating frequency, eating-disorder pathology, depression and improved weight loss were reported through a 12-month follow up in the shCBT + sibutramine group [116]. However, there are notable limitations given that sibutramine has now been withdrawn from the market due to safety concerns.

### Anorexia nervosa

Reas and Grilo [111] identified four placebo-controlled comparative studies in patients with AN, including two RCT’s investigating fluoxetine and two RCT’s trialling olanzapine, as an adjunct to inpatient and outpatient psychological interventions [111]. All four studies are notably dated (1998–2008). No significant effects were reported in the trials investigating fluoxetine as an adjunct to an inpatient program or outpatient CBT.

One of the olanzapine RCT’s [35] trialled intensive day-hospital treatment for 10 weeks in combination with either olanzapine or placebo. Thirty-four patients demonstrated significantly greater outcomes in achieving weight restoration criteria, more rapid achievement of target BMI and reduced obsessional symptoms relative to placebo [35]. However, there were no significant reductions in depression, anxiety or compulsive symptoms.

The other trial [117] was a 12-week placebo-controlled double blind RCT investigating either weekly CBT plus olanzapine or placebo in 30 patients with AN [111]. Efficacy of olanzapine as an adjunct to CBT on BMI and eating disorder psychopathology were inconclusive. Investigators reported significant increases in BMI and improvements in eating disorder psychopathology however results were not significant between olanzapine and placebo group, indicating a placebo effect [111].

## Alternative and adjunctive therapies

### Neuromodulation

Four neuromodulation techniques have been trialled for the treatment of EDs: repetitive transcranial magnetic stimulation (rTMS), transcranial direct current stimulation (tDCS), deep-brain stimulation (DBS), and neurosurgical ablation [118]. These should be reserved for the most severe cases and other procedures should be prioritised if possible. Overall, these four neuromodulatory techniques are used to target brain circuits involved in reward, mood, obsessional behaviours and inhibitory processes in an attempt to assist in the treatment of eating disorders [3, 119–121]. Neurostimulation is generally considered in treatment-refractory cases where the patient has not responded to all other types of treatment [122].

Repetitive TMS is a relatively non-invasive procedure where an electromagnetic current produced by close application of a magnet to the skull stimulates surface brain cortical regions leading to changes in neural activity. Transcranial DCS is also a relatively non-invasive procedure where the application of weak electrical currents via two electrodes attached to the scalp of a participant is used to stimulate (anodal electrode) or inhibit (cathode electrode) specific parts of the cortex. Both tDCS and rTMS appear safe for single-session application, with a risk for seizure in predisposed individuals with multiple sessions of rTMS. Similar safety issues are noted for tDCS but were more common in rTMS [123].

DBS, a more invasive, yet reversible procedure, involves the implantation of depth electrodes to deliver electrical pulses to specific areas of the brain that may ameliorate the symptoms of a disorder [118]. DBS is widely used for symptomatic relief in Parkinsons’ disease. Neurosurgical ablation is also a more invasive procedure that involves the use of Magnetic Resonance Imaging (MRI) to guide a laser to lesion small areas of the brain with abnormal structure or function without requiring traditional highly invasive cranial surgery. Unlike DBS, neurosurgical ablation, a technique developed to treat brain cancers, produces permanent and irreversible brain lesions. Therefore, this should be reserved for the most refractory cases.

#### rTMS

Systematic reviews has identified seven rTMS studies in AN, involving a total of 113 adolescents and adults. These include 2 case studies (n = 2), 2 case series (n = 7), 1 pilot study (n = 10) and 2 RCTs (n = 94). Most studies target the left dorsolateral or dorsomedial prefrontal cortex (DLPFC), which plays a major role in cognitive inhibition, emotion regulation and reward, which are all known to be affected in individuals with EDs.

The TMS case studies and case series have not demonstrated consistent weight gain although have reported lasting improvements in AN psychopathology and mood at 12-month follow-up following 20 sessions of rTMS. However, larger controlled studies of rTMS in AN report only modest results [8]. McClelland et al. [124] conducted a RCT examining a single session of rTMS versus sham stimulation in 60 patients with AN. Patients that received real rTMS (n = 28) showed an improvement in core AN symptoms (urge to restrict, feeling full, feeling fat) at end of rTMS session and 24-h follow up. However, improvements were also noted in the sham group, indicating a powerful placebo effect [124]. Dalton et al. [125] conducted a feasibility randomised controlled trial in 34 individuals with severe and enduring AN. Participants received 20 sessions (administered over 4 weeks) of rTMS to the left DLPFC or sham stimulation. At end of treatment the intervention group displayed improvements in BMI, eating disorder symptoms, quality of life and mood compared to sham group [125].

One study identified examined both BN and AN-subtypes. An open label trial conducted by Dunlop et al. [126] investigated the effectiveness of rTMS in treating binge/purge behaviours in 28 patients with AN-BP (bingepurge sub-type) or BN [126]. Patients received 20–30 sessions of dorsomedial prefrontal cortex rTMS. A significant reduction in purge frequency was observed at 4 weeks post rTMS, while there was no significant decrease in binge frequency post rTMS. However, at 6-month follow up, 16 patients (57%) had significant improvements in both binge and purge frequency [126]. There was a large degree of variance, with some participants experiencing worsening of symptoms [126]. Differences in resting state baseline corticostriatal connectivity was observed in responders relative to non-responders. Prior assessment of this connectivity via fMRI may identify candidates likely to respond favourably to rTMS treatment [126].

Eight studies involving rTMS in patients with BN (n = 148 patients) are identified by systematic reviews [118]. These include 2 case studies, 2 case series (n = 15), 1 open label (n = 28) (see above), and 3 RCTs (n = 103). All case studies were single sessions of rTMS to the dorsolateral prefrontal cortex and all reported reduced urges to eat with some case studies noting reductions in binge/purge episodes.

One RCT randomly allocated 38 individuals with BN to one session of rTMS or sham stimulation to the DLPFC and reported reduced urge to eat and binge eat at 24 h post rTMS [118]. However, Gay et al. [127] reported no therapeutic benefits of rTMS in an RCT in adults with BN and BED. No significant changes in binge/purge frequency were observed in the active group (n = 26) compared to the control group (n = 25) [127].

The other identified RCT randomised 14 adults with BN to 3 weeks of rTMS or sham stimulation. No significant effects were observed on binge/purge frequency, mood and obsessive–compulsive symptoms. Conversely, one case study in BED reported that 20 sessions of rTMS to the dlPFC significantly reduced binge frequency [118].

#### tDCS

Four studies were identified that investigated tDCS, including two studies in AN (n = 7; n = 11), one in BED (n = 30) and one in BN (n = 39), all targeting stimulation of the dorsolateral prefrontal cortex (DLPFC).

The identified studies in AN included one pilot study (n = 7) and one single blind controlled study (n = 11). The pilot study did not report any significant effects on weight gain after 10 sessions of tDCS over the DLPFC (anode left/cathode right [AL/CR]) although 3/7 patients reported significant improvements in AN psychopathology [8]. The single blind controlled study compared 18 sessions of tDCS over the DLPFC (AL/CR) to family-based treatment over 6 weeks and reported significant weight gain. However, no significant improvements were reported in AN psychopathology. Treatment related side effects included headaches, burning sensations, local itching and local redness [8].

Burges et al. [128] investigated the effects of tDCS in 30 individuals with BED and subthreshold-BED. Participants received a single 20-min session of tDCS over the DLPFC (AR/CL) and a sham stimulation. Investigators reported reduced food cravings relative to ‘sham’ tDCS sessions and reduced total food intake by 11% 5–6 h post stimulation. When participants were presented with an eating test with preferred food groups 5-6 h post stimulation, consumption decreased by 17.5% [128]. No additional follow-ups were reported. Interestingly, treatment was more efficacious in males (n = 15) compared with female (n = 26) subjects [128].

Kekic et al. [129] in a double-blind sham-controlled study investigated the effects of bilateral tDCS over the DLPFC in adults with BN. Thirty-nine participants received three sessions of tDCS in a randomised and counterbalanced order: anode right/cathode left (AR/CL), anodal left/cathode ride (AL/CR) and sham stimulation. Measures were completed during the following 24 h post stimulation. AR/CL tDCS reduced ED cognitions compared to AL/CR and sham tDCS [129]. Both active conditions reduced BN symptoms (urge to binge-eat, eating disorder related cognitions, frequency of binge eating, compensatory behaviours) compared to sham however only AR/CL improved mood compared with the sham condition [129]. There were no observed changes in food craving measured in the active compared with sham-controlled groups, contradicting results from Burgess et al. [128] on patients with BED [129]. This study further outlines the importance of optimal anode/cathode electrode montage.

#### DBS

Systematic reviews identify nine small DBS studies in AN, including case studies, case series and 2 open-label trials (see below) [8, 118, 130]. Three neuroanatomical sites have been targeted in these studies: the ventral striatum/nucleus accumbens (n = 11), subcallosal cingulate (n = 17) and the bed nucleus of the stria terminalis (BNST) (n = 2). The ventral striatum/nucleus accumbens is involved in reward processing, cognition, reinforcement learning and motivational salience; the subcallosal cingulate is involved in affect and emotion regulation; the BNST is involved in stress response, reward processing and goal directed behaviours; all of which are believed to be impacted in AN. Eight studies reported increases in BMI at follow-up with one case study showing a decrease in BMI. Normal mean BMI was achieved at final follow-up in six of the nine identified studies [8, 130].

Lipsman et al. [122, 131] conducted one of the open-label trials of DBS involving stimulation of the subcallosal cingulate (SCC) in 16 females with treatment-refractory AN. Investigators reported significant weight gain at 9 and 12-month post-surgery [122, 131]. Reduced symptoms of depression, anxiety and preoccupation with food and weight were also observed along with significant improvements in quality of life [131]. However, BMI of patients AN-BP was lower than patients AN-R (restrictive sub-type) at the 1 year follow-up. Serious adverse events were experienced in 7 participants: most related to electrolyte disturbances due to AN, but seizures (*n* = 3), and a surgical site infection (*n* = 1) were also observed. Other adverse events included nausea, air embolus, panic attack and pain at incision site [122, 131].

The other identified open-label trial [132] investigated DBS in the nucleus accumbens (NAcc) in 28 patients with treatment-refractory AN, including 13 with AN-R and 15 with AN-BP [132]. BMI significantly increased at 6-month and 2-year follow-up, with 12 (43%) normalising their BMI. Significant improvements in anxiety, mood, obsessive and compulsive symptoms were also reported at 6-month follow up and maintained at the 2-year follow up [132]. The most common side effect was short-term pain at incision site (n = 22) with no severe surgery-related adverse events were reported [132]. Similarly, to Lipsman et al. [131] investigators reported that NAcc-DBS was less effective in AN-BP compared to AN-R [132]. Further research with larger sample sizes is required to confirm this finding of differential efficacy across subtypes.

DBS of the NAcc (n = 2) and the lateral hypothalamic area (n = 3) was studied in two case studies and one case series with obese individuals (n = 3) [130]. Significant decreases in BMI, urge to binge eat and an improvement in psychiatric co-morbidities were reported. No adverse events were reported in NAcc-DBS, while patients that received lateral hypothalamic DBS reported nausea, anxiety, hot flashes and flushing [130].

DBS is an invasive procedure involving chronic implantation of depth electrodes. The lower incidence of severe adverse events in the aforementioned NAcc-DBS studies in AN (n = 11) and obesity (n = 2) suggest that this neuroanatomical site may be a safer option.

#### Neurosurgical ablation

A systematic review conducted by Murray et al. [8] identified three studies in AN involving neurosurgical ablation. This included one case study, one case series (n = 6) and a large (n = 74) open label trial. Two neuroanatomical sites targeted included the anterior limb of the internal capsule (ALIC) (n = 75) and the ventral striatum/nucleus accumbens (n = 6) [8].

Neurosurgical ablation of the ALIC in both the case study and the open label trial produced significant improvements in BMI. The case study reported significant improvements in BMI, food-related distress and ED symptoms at 3-month follow-up [8]. In the open-label trial (n = 74) significant improvements in BMI were reported at 12-month follow-up however long term-adverse events were reported in 18% of patients, included disinhibition (n = 6), memory loss (n = 3), and lethargy (n = 4) [8]. Neurosurgical ablation of the ventral striatum in the case series (n = 6) produced a rapid increase in mean BMI at 6-month and 12-month follow-up. However, no measures of psychological symptoms were reported [8].

### Yoga

Mind–body approaches, that focus on the interactions with brain, body, mind and behaviour, have been suggested as a promising intervention for eating disorders, where there is often a sense of disconnection from one’s body [133]. Research has shown that yoga can be a promising intervention for depression and anxiety and has the potential to promote embodiment and reduce factors of self-objectification, body dissatisfaction, and drive for thinness [133]. Evidently there are concerns that patients with eating disorders might turn to yoga to burn calories or suppress feelings of hunger, engaging in compulsive exercise. However, unlike most exercise, yoga aims to conserve energy and is much less strenuous. Furthermore, epidemiological studies show that regular yoga practice is associated with a reduced risk of developing an eating disorder [134].

A systematic review of eight RCTs and four observational trials totalling a sample size of 495 patients with an eating disorder (n = 330) or disordered eating/body dissatisfaction (n = 165) reported only small to moderate benefits of yoga compared to usual care on ED symptoms [134]. Only 5 studies identified met criteria for the Rapid Review and all individually reported significant improvements either in ED symptomatology or mood.

A pilot study in 20 adolescents (aged 11–18 years) with AN, BN, ARFID or OSFED, attended weekly 60–90 min yoga classes for 12 weeks. Significant improvements in anxiety, depression, weight and shape concern were reported however there were no changes in restraint and eating concerns from baseline to study completion [135].

One study examined the efficacy of 12-weeks of yoga in 90 patients with BED. Individuals were randomised 1:1 to yoga and control (on wait list for program). For the yoga group, self-reported reductions in binge eating at end of treatment were significant. Improvements on these measures were also maintained at 3-month follow-up for the yoga group. Small reductions in BMI were also reported [136].

A single-blind RCT in 30 adult females with BN or OFSED were randomized to receive 90-min of yoga two times per week for 11 weeks or control (not to perform yoga but were offered yoga after 6-month follow-up). The intervention group showed reductions in eating disorder psychopathology, restriction, eating concern and weight concern compared to the control group at 11 weeks. Improvements were further reported at 6-month follow-up [137].

One RCT assessed individualised yoga as an adjunct to standard care (n = 26) compared to standard care alone (n = 27) in 50 girls and boys aged 11–21 years with AN, BN and EDNOS. It reported that an eight-week yoga program produced a significant reduction in global ED symptomatology compared to the control group [138]. No significant differences in depression or anxiety were observed between groups [138].

Another RCT randomised yoga among inpatients (n = 38) with AN (n = 23), BN (n = 8) and EDNOS (n = 8) [133]. The yoga group (n = 20) received 50 min of yoga prior to the evening meal and the control group (n = 18) had other residential activities such as gardening or other supervised free time for 5 days. The yoga group had significantly reduced negative mood prior to meal-times however there was no significant difference in ED symptomatology [133].

### Exercise

Excessive exercise is a common symptom in eating disorders, particularly in a subgroup of patients with AN displaying highly compulsive symptomatology and behaviours [139]. This is associated with experiences of intense guilt when exercise is missed, and has been similarly observed among patients with BN [140].The care of individuals with AN has typically avoided exercise due to frequent low body weight [141]. However, given the benefits of weight bearing exercise on bone mass, muscle strength and cardiac endurance, some interventions have attempted to provide exercise therapy to patients with AN [142].

A meta-analysis including nine studies reported that supervised resistance training for patients with AN improved mood and reduced depression compared with TAU. No benefits on weight gain were observed however safety of supervised exercise in a population with AN was demonstrated [142]. A further systematic review [143] of exercise in AN patients found that despite concerns that re-introduction of exercise would encourage patients to obsessively monitor their energy intake, the opposite effect was observed [143]. Both reviews supported the benefits of resistance training to support muscle wasting and bone loss. Additionally, patients participating in nutritionally supported physical activity reported a reduction in depression and anxiety symptoms and improved feelings about food and exercise [142, 143].

A small pilot study including 36 patients with AN, BN and EDNOS indicated that sports therapy may provide benefit for individuals with eating disorders other than AN [144]. The intervention was found to reduce ‘commitment to exercise’ in the intervention group compared with controls. However, there were no significant differences in ED symptoms between groups at end of treatment. As this was a pilot study, further research is required to determine the clinical utility of sports therapy in broad ED diagnoses [144].

A pilot RCT was identified trialling an intervention which combined exercise, muscle relaxation and education in 44 patients with NES. Patients were randomised to education only (n = 14); education plus progressive muscle relaxation (PMR) therapy (n = 15) or PMR plus exercise (n = 15). Results indicated that the active component of the intervention was PMR with a significant reduction in food eaten after the evening meal in the PMR-plus-education group compared with the other treatment conditions. Exercise did not appear to have a significant effect on NES [145].

### Other alternative therapies

Two systematic reviews analysing the efficacy of alternative and complimentary therapies for patients with ED were identified. This included bright light therapy, acupuncture, eye movement desensitisation and reprocessing (EMDR), hypnosis, relaxation therapy and massage. All of the above were found to have some effect on reducing eating disorder pathology, anxiety, depressive symptoms and increase in quality in life in individuals with BN [146]. For AN, massage therapy, bright light therapy, acupuncture, EMDR, relaxation therapy reported some effects on reductions in ED pathology, depression, anxiety and an increase in quality of life [146]. Overall, however, the evidence on the relative merit and benefits of these individual therapies for individuals with an ED diagnosis is inconclusive [146].

## Discussion

The current paper has summarised the available and recent peer-reviewed evidence relating to pharmacotherapy, adjunctive and alternative therapies in the treatment of eating disorders.

Clearly the efficacy of pharmacological treatments of EDs is very limited. Despite decades of research, and widespread off-label prescribing off numerous psychotropic agents, only two pharmacotherapies are currently approved by the FDA and TGA for the treatment of EDs. These are the SSRI antidepressant fluoxetine, for BN; and the CNS stimulant, lisdexamfetamine (LDX) for BED [5, 75]. Many studies have examined potential pharmacotherapies for AN, without any single agent demonstrating consistent efficacy.

Drug trials in BED involved the largest sample populations (n > 100) of any studies examining intervention effectiveness. The CNS stimulant LDX is clearly the standout in terms of demonstrated efficacy [75, 78, 87]. LDX consistently reduces binge eating frequency as do other ADHD medications such as methylphenidate and dasotraline. Larger scale RCTs are required to better confirm efficacy of the latter agents.

Anticonvulsants including topiramate have also shown promise in the reduction of binge eating episodes and weight loss. Combination therapy of the antiepileptic zonisamide and CBT have also demonstrated promise [111, 113]. Anti-obesity drugs such as orlistat, rimonabant and liraglutide have also demonstrated efficacy in reducing binge/purge frequency and weight loss [104, 105].

The SSRI fluoxetine has been approved by the FDA and TGA for the treatment of BN. and has demonstrated efficacy in reducing binging and purging in individuals with BN, and preventing relapse [65]. Citalopram, may also help reduce binge-eating episodes and purging symptoms in BN [66]. Oxytocin and topiramate have demonstrated some clinical utility in BN for reducing binge eating frequency [52, 66].

Most pharmacotherapeutic trials in AN have investigated atypical anti-psychotics, with conflicting evidence regarding any clinical benefit of these drugs for weight restoration [20]. Low-doses of atypical-antipsychotics may be clinically useful in AN as an adjunct intervention where there is co-morbid high anxiety, obsessive-related ruminations and failure to engage in treatment [26].

Supportive evidence for SSRIs in AN are limited, yet despite this lack of demonstrable efficacy, clinicians continue to prescribe SSRIs against practice guidelines [18]. This may be due to the high co-morbidity of major depressive disorder with AN and some evidence that treatment with fluoxetine and olanzapine may help patients to remain in treatment for longer [26]. Oxytocin trials have been unable to demonstrate benefits measured by weight gain or increased calorie consumption. Oxytocin effects on attentional biases towards negative facial emotions (disgust, anger) and towards food and body image stimuli may reduce meal related stress and attention [50, 54]. The cannabinoid receptor agonist, Dronabinol, produced slight weight increase in patients with AN [45, 48, 49] and there is developing evidence for ghrelin analogues [58].

The Rapid Review also identified little evidence for efficacious pharmacological interventions for other eating disorders including ARFID, OSFED, UFED, Pica and Rumination Disorder. There is a small evidence-base relating to treatment of Night Eating Syndrome (NES) with SSRIs, with this being the only class of drug trialled in this population [6, 147].

Neuromodulatory approaches such as repetitive transcranial magnetic stimulation (rTMS), transcranial direct current stimulation (tDCS) deep-brain stimulation (DBS) and neurosurgical ablation show some preliminary promise in the treatment of EDs [8, 130].

The strongest evidence arguably pertains to the most invasive procedures: DBS and neurosurgical ablation [8, 130]. These techniques present a unique opportunity for individuals with severe and enduring AN [3]. Non-invasive neuromodulation procedures in AN tend to yield transient mixed results. While DBS is highly invasive, interestingly, no serious adverse events have been reported in NAcc-DBS [8]. Furthermore, DBS seems to be more efficacious in AN-R sub-types [8]. Although neurosurgical ablation has demonstrated promise, this remains an extreme and somewhat experimental intervention. Further large-scale trials are required to confirm efficacy of all neuromodulatory approaches.

There is some evidence for the effectiveness of yoga therapy in reducing eating disorder symptomatology in AN [133, 134, 138]. Evidence around a wide range of other complementary and alternative therapies remains inconclusive.

The purpose of the Rapid Review was to inform a translation and research strategy. This necessitated a focus on evidence that could be readily applied to the care and treatment of individuals with EDs. Due to the RR's broad-reaching and largely policy-driven intent, some limitations need to be acknowledged. Time constraints and absence of peer review prevented the inclusion of grey literature, clinical or practice guidelines, protocol papers (without results), masters’ thesis or dissertations. Due to sample sizing criteria, the research strategy was not sensitive to qualitative research. Therefore, it is likely some relevant studies were missed, especially as research into innovative pharmacotherapies and alternative therapies for the treatment of EDs is a rapidly emerging field. Further, given its focus on research conducted in countries with Western cultures and high-resource health systems, broader evidence was limited. Nevertheless, the review was able to meet its objective to identify gaps in research that may warrant further investigation, especially in the treatment of AN, BN, OFSED, UFED, Pica and rumination disorder and to provide a useful summary of the literature for those planning services or further research.

## Conclusion

The Rapid Review has identified a lack of effective pharmacotherapies, adjunctive and alternative therapies in the treatment of EDs, with the possible exception of BED. Findings suggest that more high-quality research, larger scale RCT’s and drug discovery innovation are required to better assist patients suffering with EDs.

## Supplementary Information

**Additional file 1: Figure S1**. PRISMA Diagram—Rapid Review.

**Additional file 2: Table S1**. Studies Included in the Rapid Review.

## Data Availability

Not applicable—all citations provided.

## Abbreviations

AN: Anorexia Nervosa
ARFID: Avoidant/Restrictive Intake Disorder
BMI: Body Mass Index
BED: Binge Eating Disorder
BN: Bulimia Nervosa
BWLT: Behavioural Weight Loss Therapy
CBT: Cognitive Behavioural Therapy
ED: Eating Disorder
EDNOS: Eating Disorder not otherwise specified
DBS: Deep Brain stimulation
DSM-5: Diagnostic and statistical manual of mental disorders 5^th^ edition
FDA: Food and Drug Administration
HMA: Healthcare Management Advisors
IN-OT: Intranasal Oxytocin
LDX: Lisdexamfetamine
MDD: Major Depressive Disorder
MAOi: Monoamine oxidase inhibitors
NES: Night Eating Syndrome
OFSED: Other specified feeding or eating disorder
PD: Purging Disorder
PMR: Progressive muscle relaxation
RCT: Randomised Controlled Trial
rTMS: Repetitive transcranial magnetic stimulation
RR: Rapid Review
SSRIs: Serotonergic Serotonin reuptake inhibitors
TAU: Treatment as usual
TCA: Tricyclic Antidepressants
tDCS: Transcranial Direct Stimulation
TGA: Therapeutic Goods Administration
UFED: Unspecified feeding or eating disorder

## References

[1] Beumont PJ, Touyz SW (2003). What kind of illness is anorexia nervosa?. Eur Child Adolesc Psychiatry.

[2] American Psychiatric Association, A. and A.P. Association, Diagnostic and statistical manual of mental disorders: DSM-5. 2013, Washington, DC: American psychiatric association.

[3] Treasure J (2015). New treatment approaches for severe and enduring eating disorders. Physiol Behav.

[4] Pennesi JL, Wade TD (2016). A systematic review of the existing models of disordered eating: do they inform the development of effective interventions?. Clin Psychol Rev.

[5] Himmerich H, Treasure J (2018). Psychopharmacological advances in eating disorders. Expert Rev Clin Pharmacol.

[6] Flament MF, Bissada H, Spettigue W (2012). Evidence-based pharmacotherapy of eating disorders. Int J Neuropsychopharmacol.

[7] Watson HJ, Fursland A, Byrne S (2013). Treatment engagement in eating disorders: who exits before treatment?. Int J Eat Disord.

[8] Murray SB, Strober M, Tadayonnejad R, Bari AA, Feusner JD. Neurosurgery and neuromodulation for anorexia nervosa in the 21st century: a systematic review of treatment outcomes. Eat Disord. 2022;30(1):26–53. 10.1080/10640266.2020.1790270 PMC8386186 32991247

[9] InsideOut Institute for Eating Disorders. Australian Eating Disorders Research and Translation Strategy 2021–2031. Sydney; 2021.* .*

[10] Virginia Commonwealth University. Research Guides: Rapid Review Protocol [Internet]. Rapid Review Protocol. [cited 2021 Jun 19]. Available from: https://guides.library.vcu.edu/c.php?g=240398&p=1598530.

[11] Brooks SK (2020). The psychological impact of quarantine and how to reduce it: rapid review of the evidence. Lancet.

[12] World Health Organisation. WHO | Rapid reviews to strengthen health policy and systems: a practical guide [Internet]. WHO. World Health Organization; [cited 2021 Jun 19]. Available from: http://www.who.int/alliance-hpsr/resources/publications/rapid-review-guide/en/.

[13] Canadian Agency for Drugs and Technologies in Health. About the Rapid Response Service | CADTH [Internet]. [cited 2021 Jun 19]. Available from: https://www.cadth.ca/about-cadth/what-we-do/products-services/rapid-response-service.

[14] Hamel C, Michaud A, Thuku M, Skidmore B, Stevens A, Nussbaumer-Streit B, et al. Defining rapid reviews: a systematic scoping review and thematic analysis of definitions and defining characteristics of rapid reviews. J Clin Epidemiol. 2020. 10.1016/j.jclinepi.2020.09.041 33038541

[15] Moher D (2009). Preferred reporting items for systematic reviews and meta-analyses: the PRISMA statement. PLoS Med.

[16] Aouad P (2022). Informing the development of Australia’s National Eating Disorders Research and Translation Strategy: a rapid review methodology. J Eat Disord.

[17] Hubertus H (2021). Pharmacological treatment of eating disorders, comorbid mental health problems, malnutrition and physical health consequences. Pharmacol Ther.

[18] Garner DM (2016). Psychotropic medications in adult and adolescent eating disorders: clinical practice versus evidence-based recommendations. Eat Weight Disord Stud Anorexia Bulimia Obes.

[19] Monge MC (2015). Use of psychopharmacologic medications in adolescents with restrictive eating disorders: analysis of data from the National Eating Disorder Quality Improvement Collaborative. J Adolesc Health.

[20] Balestrieri M (2013). Psychotropic drug treatment in anorexia nervosa. Search for differences in efficacy/tolerability between adolescent and mixed-age population. Eur Eat Disord Rev.

[21] Couturier J (2019). Psychotropic medication for children and adolescents with eating disorders. Child Adolesc Psychiatr Clin N Am.

[22] Gorrell S (2020). Psychotropic medication use in treatment-seeking youth with eating disorders. Eur Eat Disord Rev.

[23] Yu J (2011). A 1-year follow-up of a multi-center treatment trial of adults with anorexia nervosa. Eat Weight Disord Stud Anorexia Bulimia Obes.

[24] Mitchell JE, Roerig J, Steffen K (2013). Biological therapies for eating disorders. Int J Eat Disord.

[25] Hay PJ, Claudino AM (2012). Clinical psychopharmacology of eating disorders: a research update. Int J Neuropsychopharmacol.

[26] Márquez MC, Sánchez JM, Salazar AM, Martínez CV, Valderrama F, Rojas-Gualdrón DF. Efficacy and Safety of Antipsychotics and Antidepressants in the Treatment of Anorexia Nervosa: a Systematic Review. Rev Colomb Psiquiatr (Engl Ed). 2021. 10.1016/j.rcpeng.2022.08.007 36085125

[27] Mondraty N (2005). Randomized controlled trial of olanzapine in the treatment of cognitions in anorexia nervosa. Australas Psychiatry.

[28] Miniati M (2016). Psychopharmacological options for adult patients with anorexia nervosa. CNS Spectr.

[29] Kaye WH (2001). Double-blind placebo-controlled administration of fluoxetine in restricting-and restricting-purging-type anorexia nervosa. Biol Psychiatry.

[30] Walsh BT (2006). Fluoxetine after weight restoration in anorexia nervosa: a randomized controlled trial. JAMA.

[31] Fassino S (2002). Efficacy of citalopram in anorexia nervosa: a pilot study. Eur Neuropsychopharmacol.

[32] Holtkamp K (2005). A retrospective study of SSRI treatment in adolescent anorexia nervosa: insufficient evidence for efficacy. J Psychiatr Res.

[33] McKnight RF, Park RJ (2010). Atypical antipsychotics and anorexia nervosa: a review. Eur Eat Disord Rev Prof J Eat Disord Assoc.

[34] Lebow J (2013). The effect of atypical antipsychotic medications in individuals with anorexia nervosa: a systematic review and meta-analysis. Int J Eat Disord.

[35] Hany Bissada MD (2008). Olanzapine in the treatment of low body weight and obsessive thinking in women with anorexia nervosa: a randomized, double-blind, placebo-controlled trial. Am J Psychiatry.

[36] Frank GK (2017). The partial dopamine D2 receptor agonist aripiprazole is associated with weight gain in adolescent anorexia nervosa. Int J Eat Disord.

[37] Powers PS, Klabunde M, Kaye W (2012). Double-blind placebo-controlled trial of quetiapine in anorexia nervosa. Eur Eat Disord Rev.

[38] Attia E (2011). Olanzapine versus placebo for out-patients with anorexia nervosa. Psychol Med.

[39] Attia E (2019). Olanzapine versus placebo in adult outpatients with anorexia nervosa: a randomized clinical trial. Am J Psychiatry.

[40] Norris ML (2011). Olanzapine use for the adjunctive treatment of adolescents with anorexia nervosa. J Child Adolesc Psychopharmacol.

[41] Marzola E (2015). Atypical antipsychotics as augmentation therapy in anorexia nervosa. PLoS ONE.

[42] Court A (2010). Investigating the effectiveness, safety and tolerability of quetiapine in the treatment of anorexia nervosa in young people: a pilot study. J Psychiatr Res.

[43] Nazar BP (2017). Early response to treatment in eating disorders: a systematic review and a diagnostic test accuracy meta-analysis. Eur Eat Disord Rev.

[44] Hagman J (2011). A double-blind, placebo-controlled study of risperidone for the treatment of adolescents and young adults with anorexia nervosa: a pilot study. J Am Acad Child Adolesc Psychiatry.

[45] Andries A (2014). Dronabinol in severe, enduring anorexia nervosa: a randomized controlled trial. Int J Eat Disord.

[46] Lydecker JA, Shea M, Grilo CM (2018). Driven exercise in the absence of binge eating: implications for purging disorder. Int J Eat Disord.

[47] Andries A, Gram B, Støving RK (2015). Effect of dronabinol therapy on physical activity in anorexia nervosa: a randomised, controlled trial. Eat Weight Disord Stud Anorexia Bulimia Obes.

[48] Contreras T, Bravo-Soto GA, Rada G (2017). Do cannabinoids constitute a therapeutic alternative for anorexia nervosa?. Medwave.

[49] Rosager EV, Møller C, Sjögren M (2021). Treatment studies with cannabinoids in anorexia nervosa: a systematic review. Eat Weight Disord.

[50] Kim Y-R (2014). Intranasal oxytocin attenuates attentional bias for eating and fat shape stimuli in patients with anorexia nervosa. Psychoneuroendocrinology.

[51] Maguire S (2013). Oxytocin and anorexia nervosa: a review of the emerging literature. Eur Eat Disord Rev.

[52] Kim Y-R (2015). The impact of oxytocin on food intake and emotion recognition in patients with eating disorders: a double blind single dose within-subject cross-over design. PLoS ONE.

[53] Leppanen J, Cardi V, Ng KW, Paloyelis Y, Stein D, Tchanturia K, et al. Effects of Intranasal Oxytocin on the Interpretation and Expression of Emotions in Anorexia Nervosa. J Neuroendocrinol. 2017;29(3). 10.1111/jne.12458 PMC5363234 28140486

[54] Russell J (2018). Intranasal oxytocin in the treatment of anorexia nervosa: randomized controlled trial during re-feeding. Psychoneuroendocrinology.

[55] Misra M (2013). Impact of physiologic estrogen replacement on anxiety symptoms, body shape perception, and eating attitudes in adolescent girls with anorexia nervosa: data from a randomized controlled trial. J Clin Psychiatry.

[56] Paslakis G (2018). Prospective, randomized, double-blind, placebo-controlled phase IIa clinical trial on the effects of an estrogen-progestin combination as add-on to inpatient psychotherapy in adult female patients suffering from anorexia nervosa. BMC Psychiatry.

[57] Keating C, Tilbrook A, Kulkarni J (2011). Oestrogen: an overlooked mediator in the neuropsychopharmacology of treatment response?. Int J Neuropsychopharmacol.

[58] Fazeli PK (2018). Treatment with a ghrelin agonist in outpatient women with anorexia nervosa: a randomized clinical trial. J Clin Psychiatry.

[59] Karageorgiou V (2020). Adipokines in anorexia nervosa: a systematic review and meta-analysis. Psychoneuroendocrinology.

[60] Haleem DJ (2017). Improving therapeutics in anorexia nervosa with tryptophan. Life Sci.

[61] Achamrah N, Déchelotte P, Coëffier M (2019). New therapeutic approaches to target gut-brain axis dysfunction during anorexia nervosa. Clin Nutr Exp.

[62] Shih PB (2017). Personalized polyunsaturated fatty acids as a potential adjunctive treatment for anorexia nervosa. Prostaglandins Other Lipid Mediat.

[63] Manos BE (2018). A pilot randomized controlled trial of omega-3 fatty acid supplementation for the treatment of anxiety in adolescents with anorexia nervosa. Int J Eat Disord.

[64] Barbarich NC (2004). Use of nutritional supplements to increase the efficacy of fluoxetine in the treatment of anorexia nervosa. Int J Eat Disord.

[65] Bello NT, Yeomans BL (2018). Safety of pharmacotherapy options for bulimia nervosa and binge eating disorder. Expert Opin Drug Saf.

[66] Hay, P.J. and A.M. Claudino, Bulimia nervosa*.* BMJ clinical evidence, 2010. PMC3275326 21418667

[67] Sysko R (2010). Early response to antidepressant treatment in bulimia nervosa. Psychol Med.

[68] Hoopes SP (2003). Treatment of bulimia nervosa with topiramate in a randomized, double-blind, placebo-controlled trial, part 1: improvement in binge and purge measures. J Clin Psychiatry.

[69] Hedges DW (2003). Treatment of bulimia nervosa with topiramate in a randomized, double-blind, placebo-controlled trial, part 2: improvement in psychiatric measures. J Clin Psychiatry.

[70] Keshen AR (2021). A feasibility study evaluating lisdexamfetamine dimesylate for the treatment of adults with bulimia nervosa. Int J Eat Disord.

[71] Devlin MJ (2012). Gastric emptying and symptoms of bulimia nervosa: effect of a prokinetic agent. Physiol Behav.

[72] Hilbert A (2019). Meta-analysis of the efficacy of psychological and medical treatments for binge-eating disorder. J Consult Clin Psychol.

[73] Amodeo G (2019). Pharmacotherapeutic strategies for treating binge eating disorder. Evidence from clinical trials and implications for clinical practice. Expert Opin Pharmacother.

[74] Ramacciotti CE (2013). Therapeutic options for binge eating disorder. Eat Weight Disord.

[75] Administration, T.G. Australian public assessment report for lisdexamfetamine dimesilate. 2018: Canberra.

[76] Guerdjikova AI (2016). Lisdexamfetamine dimesylate in binge eating disorder: a placebo controlled trial. Hum Psychopharmacol Clin Exp.

[77] Fleck DE (2019). Effect of lisdexamfetamine on emotional network brain dysfunction in binge eating disorder. Psychiatry Res Neuroimaging.

[78] Brownley KA (2016). Binge-eating disorder in adults: a systematic review and meta-analysis. Ann Intern Med.

[79] Reas DL, Grilo CM (2014). Current and emerging drug treatments for binge eating disorder. Expert Opin Emerg Drugs.

[80] McElroy SL (2015). Efficacy and safety of lisdexamfetamine for treatment of adults with moderate to severe binge-eating disorder: a randomized clinical trial. JAMA Psychiat.

[81] McElroy SL (2016). Lisdexamfetamine dimesylate for adults with moderate to severe binge eating disorder: results of two pivotal phase 3 randomized controlled trials. Neuropsychopharmacology.

[82] McElroy SL (2017). Time course of the effects of lisdexamfetamine dimesylate in two phase 3, randomized, double-blind, placebo-controlled trials in adults with binge-eating disorder. Int J Eat Disord.

[83] Gasior M (2017). A phase 3, multicenter, open-label, 12-month extension safety and tolerability trial of lisdexamfetamine dimesylate in adults with binge eating disorder. J Clin Psychopharmacol.

[84] Hudson JI (2017). Efficacy of lisdexamfetamine in adults with moderate to severe binge-eating disorder: a randomized clinical trial. JAMA Psychiat.

[85] Ward K, Citrome L (2018). Lisdexamfetamine: chemistry, pharmacodynamics, pharmacokinetics, and clinical efficacy, safety, and tolerability in the treatment of binge eating disorder. Expert Opin Drug Metab Toxicol.

[86] Kornstein SG (2019). Clinical characteristics and treatment response to lisdexamfetamine dimesylate versus placebo in adults with binge eating disorder: analysis by gender and age. J Clin Psychiatry.

[87] Citrome L (2015). Lisdexamfetamine for binge eating disorder in adults: a systematic review of the efficacy and safety profile for this newly approved indication—what is the number needed to treat, number needed to harm and likelihood to be helped or harmed?. Int J Clin Pract.

[88] Agh T (2016). The cost effectiveness of lisdexamfetamine dimesylate for the treatment of binge eating disorder in the USA. Clin Drug Investig.

[89] Quilty LC (2019). A randomized comparison of long acting methylphenidate and cognitive behavioral therapy in the treatment of binge eating disorder. Psychiatry Res.

[90] Davis C (2016). Sex differences in subjective and objective responses to a stimulant medication (methylphenidate): comparisons between overweight/obese adults with and without binge-eating disorder. Int J Eat Disord.

[91] McElroy SL (2015). Armodafinil in binge eating disorder: a randomized, placebo-controlled trial. Int Clin Psychopharmacol.

[92] Grilo CM (2021). Efficacy and safety of dasotraline in adults with binge-eating disorder: a randomized, placebo-controlled, fixed-dose clinical trial. CNS Spectr.

[93] McElroy SL (2020). Efficacy and safety of dasotraline in adults with binge-eating disorder: a randomized, placebo-controlled, flexible-dose clinical trial. J Clin Psychiatry.

[94] Grant JE (2019). A double-blind, placebo-controlled study of vortioxetine in the treatment of binge-eating disorder. Int J Eat Disord.

[95] White MA, Grilo CM (2013). Bupropion for overweight women with binge-eating disorder: a randomized, double-blind, placebo-controlled trial. J Clin Psychiatry.

[96] Chamberlain SR (2012). Effects of mu opioid receptor antagonism on cognition in obese binge-eating individuals. Psychopharmacology.

[97] McElroy SL (2013). A placebo-controlled pilot study of the novel opioid receptor antagonist ALKS-33 in binge eating disorder. Int J Eat Disord.

[98] Guerdjikova AI (2017). Concurrent improvement in both binge eating and depressive symptoms with naltrexone/bupropion therapy in overweight or obese subjects with major depressive disorder in an open-label, uncontrolled study. Adv Ther.

[99] Grilo CM (2021). Naltrexone + bupropion combination for the treatment of binge-eating disorder with obesity: a randomized, controlled pilot study. Clin Therap.

[100] McElroy SL (2011). Acamprosate in the treatment of binge eating disorder: a placebo-controlled trial. Int J Eat Disord.

[101] Nourredine M (2021). Efficacy and safety of topiramate in binge eating disorder: a systematic review and meta-analysis. CNS Spectr.

[102] Sala M (2017). A double-blind, randomized pilot trial of chromium picolinate for overweight individuals with binge-eating disorder: effects on glucose regulation. J Diet Suppl.

[103] Brownley KA (2013). A double-blind, randomized pilot trial of chromium picolinate for binge eating disorder: results of the Binge Eating and Chromium (BEACh) study. J Psychosom Res.

[104] Robert SA (2015). Improvement in binge eating in non-diabetic obese individuals after 3 months of treatment with liraglutide: a pilot study. Obes Res Clin Pract.

[105] Pataky Z (2013). Efficacy of rimonabant in obese patients with binge eating disorder. Exp Clin Endocrinol Diabetes.

[106] Vander Wal JS (2012). Night eating syndrome: a critical review of the literature. Clin Psychol Rev.

[107] Vander Wal JS (2012). Escitalopram for treatment of night eating syndrome: a 12-week, randomized, placebo-controlled trial. J Clin Psychopharmacol.

[108] Allison KC (2013). An open-label efficacy trial of escitalopram for night eating syndrome. Eat Behav.

[109] Allison KC, Tarves E (2011). Treatment of night eating syndrome. Psychiatr Clin N Am.

[110] Matsui K (2021). The efficacy of add-on ramelteon and subsequent dose reduction in benzodiazepine derivatives/Z-drugs for the treatment of sleep-related eating disorder and night eating syndrome: a retrospective analysis of consecutive patients. J Clin Sleep Med.

[111] Reas DL, Grilo CM (2021). Psychotherapy and medications for eating disorders: better together?. Clin Ther.

[112] Larranaga A (2014). Comparative study of cognitive-behavioral psychotherapy and nutritional support in patients with different types of eating disorders. Med Clin.

[113] Grilo CM, Reas DL, Mitchell JE (2016). Combining pharmacological and psychological treatments for binge eating disorder: current status, limitations, and future directions. Curr Psychiatry Rep.

[114] Grilo CM (2012). 12-month follow-up of fluoxetine and cognitive behavioral therapy for binge eating disorder. J Consult Clin Psychol.

[115] Blomquist KK, Grilo CM (2011). Predictive significance of changes in dietary restraint in obese patients with binge eating disorder during treatment. Int J Eat Disord.

[116] Grilo CM (2015). Predicting meaningful outcomes to medication and self-help treatments for binge-eating disorder in primary care: the significance of early rapid response. J Consult Clin Psychol.

[117] Brambilla F (2007). Olanzapine therapy in anorexia nervosa: psychobiological effects. Int Clin Psychopharmacol.

[118] Dalton B (2018). Neurostimulation in clinical and sub-clinical eating disorders: a systematic update of the literature. Curr Neuropharmacol.

[119] Rachid F (2018). Repetitive transcranial magnetic stimulation in the treatment of eating disorders: a review of safety and efficacy. Psychiatry Res.

[120] Val-Laillet D (2015). Neuroimaging and neuromodulation approaches to study eating behavior and prevent and treat eating disorders and obesity. NeuroImage Clin.

[121] Park RJ, Godier LR, Cowdrey FA (2014). Hungry for reward: how can neuroscience inform the development of treatment for Anorexia Nervosa?. Behav Res Ther.

[122] Lipsman N (2017). Deep brain stimulation of the subcallosal cingulate for treatment-refractory anorexia nervosa: 1 year follow-up of an open-label trial. The Lancet Psychiatry.

[123] Hall PA, Vincent CM, Burhan AM (2018). Non-invasive brain stimulation for food cravings, consumption, and disorders of eating: a review of methods, findings and controversies. Appetite.

[124] McClelland J (2016). A randomised controlled trial of neuronavigated repetitive transcranial magnetic stimulation (rTMS) in anorexia nervosa. PLoS ONE.

[125] Bethan D (2018). Randomised controlled feasibility trial of real versus sham repetitive transcranial magnetic stimulation treatment in adults with severe and enduring anorexia nervosa: the TIARA study. BMJ Open.

[126] Dunlop K (2015). Increases in frontostriatal connectivity are associated with response to dorsomedial repetitive transcranial magnetic stimulation in refractory binge/purge behaviors. NeuroImage Clin.

[127] Gay A (2016). A lack of clinical effect of high-frequency r TMS to dorsolateral prefrontal cortex on bulimic symptoms: a randomised, double‐blind trial. Eur Eat Disord Rev.

[128] Burgess EE (2016). Effects of transcranial direct current stimulation (tDCS) on binge-eating disorder. Int J Eat Disord.

[129] Kekic M (2017). Single-session transcranial direct current stimulation temporarily improves symptoms, mood, and self-regulatory control in bulimia nervosa: a randomised controlled trial. PLoS ONE.

[130] Potes MI (2021). The utility of deep brain stimulation surgery for treating eating disorders: a systematic review. Surg Neurol Int.

[131] Lipsman N (2013). Subcallosal cingulate deep brain stimulation for treatment-refractory anorexia nervosa: a phase 1 pilot trial. The Lancet.

[132] Liu W (2020). Deep brain stimulation of the nucleus accumbens for treatment-refractory anorexia nervosa: a long-term follow-up study. Brain Stimul.

[133] Pacanowski CR (2017). Yoga in the treatment of eating disorders within a residential program: a randomized controlled trial. Eat Disord.

[134] Ostermann T (2019). Effects of yoga on eating disorders—a systematic review. Complement Ther Med.

[135] Hall A (2016). Use of yoga in outpatient eating disorder treatment: a pilot study. J Eat Disord.

[136] McIver S, O’Halloran P, McGartland M (2009). Yoga as a treatment for binge eating disorder: a preliminary study. Complement Ther Med.

[137] Karlsen KE (2018). Effect of yoga in the treatment of eating disorders: a single-blinded randomized controlled trial with 6-months follow-up. Int J Yoga.

[138] Carei TR (2010). Randomized controlled clinical trial of yoga in the treatment of eating disorders. J Adolesc Health.

[139] Sauchelli S (2015). Physical activity in anorexia nervosa: How relevant is it to therapy response?. Eur Psychiatry.

[140] Mond JM, Calogero RM (2009). Excessive exercise in eating disorder patients and in healthy women. Aust N Z J Psychiatry.

[141] Ibrahim A, Cutinha D, Ayton A (2019). What is the evidence for using bed rest as part of hospital treatment of severe anorexia nervosa?. Evid Based Ment Health.

[142] Ng L, Ng D, Wong W (2013). Is supervised exercise training safe in patients with anorexia nervosa? A meta-analysis. Physiotherapy.

[143] Moola FJ, Gairdner SE, Amara CE (2013). Exercise in the care of patients with anorexia nervosa: a systematic review of the literature. Ment Health Phys Act.

[144] Schlegel S (2015). The Freiburg sport therapy program for eating disordered outpatients: a pilot study. Eat Weight Disord Stud Anorexia Bulimia Obes.

[145] Vander Wal JS (2015). Education, progressive muscle relaxation therapy, and exercise for the treatment of night eating syndrome. A pilot study. Appetite.

[146] Fogarty S, Smith CA, Hay P (2016). The role of complementary and alternative medicine in the treatment of eating disorders: a systematic review. Eat Behav.

[147] McElroy SL (2015). Psychopharmacologic treatment of eating disorders: emerging findings. Curr Psychiatry Rep.

