# Supplementary material for: Pharmacotherapy, alternative and adjunctive therapies for eating disorders: findings from a rapid review
Source: J Eat Disord. 2023 Jul 6;11:112. doi: 10.1186/s40337-023-00833-9 (PMC10327007; doi:10.1186/s40337-023-00833-9)
Supplement: Supplementary file 1 — Additional file 1: Figure S1. PRISMA Diagram—Rapid Review. [file 40337_2023_833_MOESM1_ESM.docx]

Additional File 1

Figure 1. PRISMA Diagram – Rapid Review

Articles identified through database searching

(n=17,757)

Articles identified through links and reference lists

(n=36)

Articles after duplicates removed

(n=9,260)

Articles screened through assessment of abstract/title

(n=9,260)

Excluded

(n=7,292)

Full text articles assessed for eligibility

(n=1,968)

Excluded

(n=660)

Expert research collaborative requested articles

(n=12)

Articles included in Rapid Evidence Review

(n=1,320)

Identification

Screening

Eligibility

Included
