# Supplementary material for: Pharmacotherapy, alternative and adjunctive therapies for eating disorders: findings from a rapid review
Source: J Eat Disord. 2023 Jul 6;11:112. doi: 10.1186/s40337-023-00833-9 (PMC10327007; doi:10.1186/s40337-023-00833-9)
Supplement: Supplementary file 2 — Additional file 2: Table S1. Studies Included in the Rapid Review. [file 40337_2023_833_MOESM2_ESM.docx]

Additional file 2

Table 1. Studies Included in the Rapid Review

| **Author, Year** | **Country** | **N** | **Population** | **Aim** | **Design** | **Outcome measure** |
| --- | --- | --- | --- | --- | --- | --- |
| Achamrah, Dechelotte and Coeffier, 2019 | N/A | N/A | N/A | To review the potential effects of physical activity on gut-brain axis during refeeding, and propose future combined strategies for AN treatment | Review (Narrative) | Effect on gut-brain axis during refeeding |
| Agh et al., 2016 | USA | N/A | N/A | To evaluate the cost effectiveness of LDX versus no pharmacotherapy (NPT) in adults with BED from a USA healthcare payer’s perspective. | Modelling (Statistical) | Quality adjusted life years, total therapy cost, incremental cost-effectiveness ratio |
| Allison and Tarves, 2011 | N/A | N/A | N/A | To outline the basic features of the syndrome and summarize the treatment approaches that have been developed for NES. | Review (Narrative) | No direct measures - outlines various treatments for NES (including Abbreviated PMR, Sertraline, CBT, PMR, Phototherapy) |
| Allison et al., 2013 | USA | 31 | Community (Adult, All Genders) | To expand the understanding of the efficacy of escitalopram in the current trial. | Quasi-experimental (intervention) | Percent of daily intake after the evening meal, number of nocturnal ingestions (NI)/week, weight, total awakenings/week, mood, and quality of life. |
| Amodeo et al., 2019 | Worldwide | N/A | Community (Adult, All Genders) | To review the main pharmacological treatments for BED and provides an expert opinion based on the available evidence and on the authors’ clinical experience with patients affected by BED. | Review (Narrative) | Weight reduction and reduction of binge eating |
| Andries et al., 2014 | Denmark | 25 | outpatient (adult, women) | To investigate the effects of treatment with a synthetic cannabinoid agonist (dronabinol) on body weight and eating disorder-related psychopathological personality traits in women with severe, enduring AN. | RCT (cross-over) | Weight change, eating disorder inventory (EDI 2) |
| Andries, Gram and Stoving, 2014 | Denmark | 24 | Outpatient (Adult, Women) | To (1) assess the effect of dronabinol-a synthetic cannabinoid agonist-on physical activity in patients with chronic and stable AN, and to (2) unravel the role of leptin and cortisol in this process. | RCT (cross-over) | Physical activity, body weight, leptin and urinary free cortisol excretion |
| Attia et al., 2011 | USA, Canada | 23 | Outpatient (Adult, All Genders) | To determine whether olanzapine is superior to placebo in increasing body mass index (BMI) and improving psychological symptoms in out-patients with AN. | RCT | BMI, psychological symptoms Yale–Brown–Cornell Eating Disorders Scale (YBC-EDS); Positive and Negative and Syndrome Scale (PANSS). |
| Attia et al., 2019 | USA | 152 | Outpatient (Adult, All Genders) | To evaluate the benefits of olanzapine compared with placebo for adult outpatients with anorexia nervosa. | RCT | BMI, YBC-EDS, EDE, center for epidemiologic studies depression scale (CES-D), anxiety, clinical global impressions scale) severity (CGI). |
| Balestrieri et al., 2013 | Worldwide | 22 | Community (Adult, All Genders) | To review the literature on the use of antidepressants and antipsychotics in adolescents with anorexia nervosa, comparing the efficacy and tolerability in this population with those reported in trials with patients not selected by age. | Review (Systematic) | BMI, eating disorder symptoms, functional impairment |
| Bello and Yeomans, 2018 | Worldwide | N/A | N/A | To examine the efficacy and safety profile of the FDA-approved medica- tions for the treatment of BN and BED. This will include the evaluation of fluoxetine for BN, and lisdexamfetamine for BED. Safety information will be review from randomized control trials (RCT), open label trials, and case reports. | Review (Narrative) | Side effects, safety and well-toleratedness |
| Blomquist and Grilo, 2011 | USA | 50 | Inpatient (Adult, All Genders) | To examine whether changes in different aspects of dietary restraint in obese patients with BED participating in a treatment study predict outcomes. | RCT | “Abstinence” from binge eating, “5% weight loss” from baseline weight |
| Brownley et al., 2013 | USA | 24 | Community (Adult, All Genders) | To evaluate the the usefulness of chromium in the treatment of BED | RCT | Mean change in binge frequency and related psychopathology, weight, symptoms of depression, and fasting glucose. |
| Burgess et al., 2016 | England | 30 | Community (Adult, All Genders) | To investigate the effect of transcranial direct current stimulation (tDCS) on food craving, intake, binge eating desire, and binge eating frequency in individuals with BED. | RCT | Food craving, food intake, binge eating frequency, binge eating desire. |
| Carei et al., 2010 | USA | 54 | Community (Adolescents, All Genders) | To assess the effect of individualized yoga treatment on eating disorder outcomes among adolescents receiving outpatient care for diagnosed eating disorders (anorexia nervosa, bulimia nervosa, eating disorder not otherwise specified). | RCT | EDE, BMI, Beck Depression Inventory (BDI-II), State-Trait Anxiety Inventory (STAI), Food Preoccupation questionnaire. |
| Cassioli et al., 2020 | Worldwide | 19 | Community (Adult, All Genders) | To perform an updated systematic review and meta-analysis of published randomized controlled trials investigating psychopharmacological treatment in acute-phase AN. | Systematic Review/ Meta-Analysis (combined) | Effect of psychoactive drugs on BMI, psychopathological outcomes. |
| Chamberlain et al., 2012 | UK | 63 | Community (Adult, All Genders) | To objectively assess the cognitive effects of a mu opioid receptor antagonist in obese individuals with binge-eating symptoms. | RCT | Processing bias for food stimuli (visual dot prob with 500- and 2000-ms stimulus presentations, and food strop tasks). Other distinct cognitive functions: N-back working memory, sustained attention, and power of attention tasks. |
| Citrome, 2015 | Worldwide | 3 | Community (Adult, All Genders) | To describe the efficacy and safety of LDX for the treatment of BED. | Review (Systematic) | BED symptoms (measured by binge-eating days/week) |
| Contreras et al., 2017 | Worldwide | 2 | Inpatient (Adult, All Genders) | To systematically review the effect of cannabinoids as a therapeutic alternative for anorexia nervosa | Review (Systematic) | Weight, symptoms of AN |
| Court et al., 2010 | Australia | 33 | Outpatient (Young People, All Genders) | To investigate the safety and tolerability of the atypical antipsychotic quetiapine in anorexia nervosa patients, and to determine the effect of quetiapine treatment on anorexic psychopathology and other key outcome measures including weight and body image, | RCT | EDI-2. CES-D, Mood and anxiety symptom questionnaire (MASQ), personal wellbeing index (PWI), The managing affect and differences scale (MADS) |
| Courtier et al., 2019 | Worldwide | N/A | Community (Mixed Cohort, All Genders) | To review the literature on the efficacy of psychotropic medications used exclusively to treat children and adolescents with primary EDs | Review (Critical) | Efficacy of psychotropic medications for children and adolescents |
| Dalton et al., 2018 | Worldwide | 32 | Community (Adult, All Genders) | To provide an update of the literature examining the effects of DBS, rTMS and tDCS on eating behaviours, body weight and associated symptoms in people with EDs and relevant analogue populations. | Review (Systematic) | Effects of neurostimulation techniques on eating and weight-related outcomes in people with EDs. |
| Dalton et al., 2020 | UK | 30 | Community (Adult, All Genders) | To assess longer-term effects of rTMS on ED symptoms and general psychopathology in the TIARA (Transcranial magnetic stimulation and imaging in anorexia nervosa: the TIARA study) participants in an open follow-up 18-months post-randomisation. | RCT | Changes in BMI, eating disorder examination questionnaire (EDE-Q), clinical impairment assessment questionnaire (CIA), Depression anxiety stress scales (DASS-21) |
| Davis et al., 2016 | USA | 198 | Community (Adult, All Genders) | To examine sex differences in response to a single dose of a psychomotor-stimulant medication (methylphenidate: MP) and to assess whether expected differences were moderated by BED status. | RCT (cross-over) | Emotional/mood ratings (via subjective rating scales), appetite ratings, food cravings, snack-food consumption |
| Devlin et al., 2012 | USA | 56 | Community (Adult, Women) | To test these hypotheses as well as the efficacy of the prokinetic agent erythromycin in patients with BN. hypothesis - Previous studies have suggested that delayed gastric emptying and abnormal postprandial release of hormones that influence satiation, particularly cholecystokinin (CCK), may play an important role in the pathophysiology of BN. | RCT | primary: gastric emptying and hormone levels, secondary: BDI score, restraint and hunger score, binge and vomit frequency |
| Dunlop et al., 2015 | Canada | 28 | Community (Adult, All Genders) | To characterize and distinguish the neural activity of subpopulations of patients showing improvement versus non-improvement in this population. | Quasi-experimental (intervention) | Weekly frequency of binge and purge episodes, EDE scores |
| Fazeli et al., 2018 | USA | 22 | Outpatient (adult, women) | To assess the effects of relamorelin on weight gain and gastric emptying in women with AN | RCT | Weight, plasma ghrelin levels, gastric emptying, depression, resting expenditure |
| Flament, Bissada and Spettigue, 2012 | Worldwide | N/A | Community (Mixed Cohort, All Genders) | To review scientific evidence for efficacy and safety of pharmacotherapy in adults or children with an eating disorder. | Review (Critical) | Efficacy of drugs (pharmacotherapy) in reducing ED symptomology |
| Fleck et al., 2019 | USA | 20 | Community (Adult, Women) | To examine the effects of LDX treatment on ventral prefrontal cortex (VPFC) and striatal brain activation in BED. | Quasi-experimental (intervention) | CGI-I, compulsivity, bevahiour evaluation scale (BES), BMI, number of binge days per week |
| Fogarty, Smith and Hay, 2016 | Worldwide | 16 | Community (Adult, All Genders) | To examine the role of CAM therapies in the treatment of eating disorders. | Review (Systematic) | Primary outcomes: improvements in eating psychopathology (e.g. binge eating, restriction, drive for thinness, shape and eating concerns) or improvements in general psychopathology (weight, anxiety, depression, quality of life). Secondary outcomes: side-effects or negative effects and patient satisfaction. |
| Garner et al., 2016 | USA | 501 | Inpatient (Adult, All Genders) | To determine the frequency of psychotropic use in clinical practice and ascertain the degree to which it conforms to well established evidence-based recommendations for both AN and BN | Cross-Sectional | BMI, Self-reports (EAT-26) |
| Gasior et al., 2015 | USA | 604 | Community (Adult, All Genders) | To assess the long-term safety and tolerability of LDX in adults with BED. | Quasi-experimental (intervention) | Treatment emergent adverse events (TEAE), Vital Sign, and ECG: Safety Analysis Set: assessments of vital signs, adverse events (AEs), the 12-lead ECG, and the Colombia Suicide Severity Rating Scale (C-SSRS) |
| Gay et al., 2016 | France | 47 | Outpatient (Adult, Women) | To investigate the safety and therapeutic efficacy of an adjunct high-frequency rTMS programme targeting the left DLPFC. | RCT | Primary outcomes: number of binge episodes in the 15 days before the final visit. Secondary outcomes: characteristics of the binge episodes, the number of vomiting episodes before the final visit, the mood at the final visit, the modification in the outcomes before and after the baseline and final assessments. |
| Gorrell et al., 2021 | USA | 604 | Outpatient (Young People, All Genders) | To (1) describe the prevalence and patterns of psychotropic medication use in a large treatment-seeking sample of youth presenting with EDs to a specialist outpatient service at an academic medical centre in the United States, and (2) examine child and family characteristics associated with psychotropic medication use. | Cross-Sectional | Assessment of medication use (medication name and dosage), Eating disorder examination (EDE) |
| Grant et al., 2019 | USA | 80 | Community (Adult, All Genders) | To examine the efficacy and safety of vortioxetine compared to placebo in adults with moderate to severe BED | RCT | Assessment of BED: BE diary, CGI-I and CGI-S, Three-factor eating questionnaire, Yale-Brown Obsessive Compulsive Scale modified for Binge Eating (Y-BOCS-BE), weight. Baseline psychiatric evaluation: MINI International Neuropsychiatric interview. Baseline & follow-up assessments: urine pregnancy test and urine drug screen, psychiatric evaluations (HAM-Depression, HAM-Anxiety, SDS, C0SSRS). |
| Grilo et al., 2012 | USA | 81 | Community (Adult, All Genders) | To examine the longer term effects of fluoxetine and cognitive behavioural therapy (CBT) either with fluoxetine (CBT + fluoxetine) or with placebo (CBT + placebo) for BED through 12-month follow-up after completing treatments. | RCT | Remission from binge eating, continuous measures of binge-eating frequency, eating disorder psychopathology, depression, and weight. |
| Grilo et al., 2015 | USA | 104 | Inpatient (Adult, All Genders) | To examine rapid response among obese patients with BED in a randomized clinical trial testing antiobesity medication and self-help cognitive–behavioural therapy (shCBT), alone and in combination, in primary-care settings. | RCT | EDE scores, EDE-Q scores (to determine rapid response), Beck Depression Inventory (BDI), weight and height (to calculate BMI). |
| Grilo et al., 2021 | USA | 22 | Community (Adult, All Genders) | To evaluate the acute effects of naltrexone + bupropion (NB) on BED with obesity and examine the longer-term effects through 6-month follow-up after the discontinuation of medication. | RCT | Primary outcomes: Changes from baseline in binge-eating frequency, percentage weight. Secondary outcomes: Changes in eating-disorder psychopathology, depression. |
| Grilo, Masheb and Crosby, 2012 | USA | 108 | Inpatient (Adult, All Genders) | To examine predictors and moderators of response to cognitive behavioral therapy (CBT) and medication treatments for BED | RCT | Primary outcome: Remission from binge eating. Secondary dimensional outcomes: binge-eating frequency, eating disorder psychopathology, depression, and body mass index. |
| Grilo, Reas and Mitchell, 2016 | Worldwide | N/A | N/A | To provide a brief state-of-the-art review of the major and recent findings, with an emphasis on developments over the past 3 years. | Review (Narrative) | Summary of RCTs for combined psychological and pharmacological treatment of BED |
| Guardia et al., 2011 | Worldwide | N/A | N/A | To review published data on the efficacy and safety of drugs targeting the GABA and glutamate modulation pathways for the treatment of BED, BN and NES | Review (Other) | binge eating, craving and weight gain |
| Guerdjikova et al., 2016 | USA | 50 | Community (Adult, All Genders) | To evaluate lisdexamfetamine dimesylate (LDX) in the treatment of binge eating disorder (BED). | RCT | Binge eating days/week, binge eating episodes/week, CGI-Scale, yale-brown obsessive compulsive scale ( Y-BOSC), weight, BMI, fasting triglyceride level |
| Guerdjikova et al., 2017 | USA | 25 | Community (Adult, Women) | To assess the effect of NB in reducing binge eating symptoms associated with MDD and overweight/obesity | Quasi-experimental (intervention) | Body weight, MADRS, DES, Control of eating questionnaire (CoEQ), binge eating scale (BES), Inventory of Depressive Symptomatology self report ( IDS-SR) |
| Guido et al., 2017 | Worldwide | 106 | Community (Adolescents, All Genders) | To assess the use of dopamine D2 receptor partial agonists in treating AN. | Review (Other) | Change in BMI |
| Hagman et al., 2011 | USA | 40 | outpatient (young adult, women) | To evaluate the safety and efficacy of risperidone for the treatment of anorexia nervosa. | RCT | BMI, EDI-2 drive thinness, multidimensional anxiety scale for children (MASC), body image software, point of subjective equality |
| Haleem, 2017 | N/A | N/A | N/A | To evaluate and document a potential importance of tryptophan supplementation in improving therapeutics in AN patients | Review (Other) | Importance of tryptophan supplementation |
| Hall, Vincent and Burhan, 2017 | Worldwide | N/A | Community (Adult, All Genders) | To describe the state of the human research literature pertaining to the use of non-invasive brain stimulation (NIBS) procedures for modulating food cravings, food consumption, and treating disorders of eating (i.e., obesity, bulimia nervosa, and anorexia nervosa). | Review (Narrative) | Food cravings, food consumption, treating disorders of eating |
| Hay & Claudino, 2012 | Worldwide | N/A | Community (Adult, All Genders) | To review the use of psychopharmacological agents in eating disorders | Review (Critical) | Clinical effects of medication |
| Hay and Claudino, 2010 | Worldwide | 27 | Community (Adult, Women) | To examine the effects of treatments and discontinuing treatment in adults with bulimia nervosa | Review (Systematic) | Frequency of vomiting, remission, depression |
| Hilbert et al., 2019 | Worldwide | 81 | Community (Adult, Women) | To provide a comprehensive meta-analysis on the efficacy of psychological and medical treatments for BED, including those targeting weight loss. | Meta-Analysis | Primary outcomes: number of binge-eating episodes, abstinence from binge eating. Secondary outcomes: eating disorder psychopathology was operationalized through attitudes regarding eating behaviour and body image, and general psychopathology was operationalized through measures of depression, body weight and BMI. |
| Himmerich and Treasure, 2017 | Worldwide | N/A | Community (Adult, All Genders) | To summarize novel clinical and epidemiological data, outline genetic and neurobiological advances, and comment on possible future translational and personalized approaches. To bring together the results of pharmacological research, brain imaging, and RCTs to develop a hypothetical model of where drugs for EDs act in the brain. | Review (Other) | What drugs do, and where drugs act in brains of ED patients |
| Hudson et al., 2017 | USA | 418 | Community (Adult, All Genders) | To assess lisdexamfetamine dimesylate maintenance of efficacy in adults with moderate to severe binge-eating disorder. | RCT | Time to relapse (measured by Binge-eating days/week, and CGI-S Scores) |
| Karageorgiou et al., 2019 | Worldwide | 152 | Community (Adult, All Genders) | To (1) synthesize the available evidence on potential differences between AN patients and controls with regards to adipokine measurements (namely, leptin, adiponectin, resistin, soluble leptin receptor, visfatin, vaspin and omentin); (2) estimate the potential differences between constitutionally thin (CT) subjects and AN patients, and (3) present the available evidence with regards to biomarker efficacy of adipokines in AN. | Systematic Review/ Meta-Analysis (combined) | Treatment outcomes: time to BMI restoration, better response of dietary behaviour in response to therapy, psychological symptoms |
| Keating, Tilbrook and Kulkarni, 2011 | Worldwide | N/A | Community (Mixed Cohort, female) | To look at the relationship between primarily major depression (MD), AN, Oestrogen, and secondarily 5-HT, HPA axis, MD, mood, and stress. | Review (Narrative) | Stress, mood, ED symptoms |
| Kekic et al., 2017 | England | 39 | Community (Adult, All Genders) | To investigate the effects of bilateral tDCS over the DLPFC in adults with BN. | RCT | Primary outcome: urge to binge-eat (VAS). Secondary outcomes: Mizes Eating Disorder Cognition Questionnaire-Revised (MEDCQ-R), Food Challenge Task (FCT), TD task, Profie of Mood States (POMS), Positive and Ngetaive Affect Shcedule (PANAS), and tolerability, acceptability, and blinding of tDCS. |
| Keshen et al., 2021 | Canada | 18 | Community (Adult, All Genders) | To collect information regarding the feasibility, safety, and potential efficacy of LDX as a treatment for adults with BN. | Quasi-experimental (intervention) | Enrolment rate, dropout rate, safety outcomes, and eating disorder symptom change |
| Khalil, Souaiby and Fares, 2017 | Worldwide | N/A | Community (Adult, All Genders) | To review the evidence suggesting a causal relationship among the HPA axis and the limbic system in the symptoms of AN, and link findings to the potential efficacy of the progesterone and type II glucocorticoid receptor antagonist mifepristone (RU486) in modulating the hyperactivation of the HPA axis and improving AN symptom. | Review (Critical) | Symptoms of AN - HPA axis and limbic system link for reducing symptoms |
| Kim et al., 2014 | Korea | 64 | Community (Adult, Women) | To broadly examine whether oxytocin might be beneficial in the short term. | RCT | EDE, EDE-Q, Autism spectrum quotient (AQ), BDI, STAI |
| Kim et al., 2015 | Korea | 102 | Outpatient and Community (Adult, Women) | To examine the impact of intranasal oxytocin on consummatory behaviour and emotional recognition in patients with AN and BN in comparison to healthy controls. | RCT (cross-over) | Appetite, calorie consumption, emotion recognition sensitivity, consummatory behaviour |
| Kisihi et al., 2012 | Worldwide | 8 | Inpatient (Adult, All Genders) | To assess the utility of antipsychotics for weight gain and improvement of illness-related psychopathology in patients with anorexia nervosa. | Systematic Review/ Meta-Analysis (combined) | Body weight (expressed as SMD between baseline and endpoint BMI, endpoint BMI, or daily weight change) |
| Kornstein et al., 2019 | USA | 745 | Outpatient (Adult, All Genders) | To describe clinical characteristics and LDX treatment effects based on gender and age - in adults diagnosed with moderate to severe binge eating disorder (BED) | RCT | Treatment efficacy, safety, tolerability, of LDX: age, gender, - vital signs, TEAE, binge eating days/ week, CGI, |
| Larranaga et al., 2014 | Spain | 74 | Community (Adult, All Genders) | To compare response to Cognitive Behavioral Therapy in patients with different clinical forms of eating disorders | RCT | Recovery rate, rate of improvements, rate of patients with poor outcomes, predictive outcomes |
| Lebow et al., 2012 | Worldwide | N/A | Mixed (All Genders) | To estimate the influence of atypical antipsychotics on BMI, eating disorder, and psychiatric symptoms in individuals with AN. | Systematic Review/ Meta-Analysis (combined) | BMI, EDI, depression, anxiety, weight |
| Leppanen et al., 2017 | UK | 39 | Mixed (Adult, Women) | To investigate the effects of a single dose of intranasal oxytocin (40 IU) on a standard laboratory smoothie challenge, and on salivary cortisol, anxiety, and attentional bias towards food images before and after the smoothie challenge in AN and HC participants | RCT (cross-over) | Attentional avoidance of food images, smoothie consumption, anxiety, salivary control, attentional bias (food images). |
| Lipsman et al., 2013 | Canada | 6 | Community (Adult, All Genders) | To assess the safety of DBS to modulate the activity of limbic circuits and to examine how this might affect the clinical features of anorexia nervosa. | Longitudinal (<5yr) | Primary outcome: adverse events associated with surgery or stimulation. Secondary outcomes: repeat psychometric assessments, BMI, and neuroimaging investigations at various intervals. |
| Lipsman et al., 2017 | Canada | 6 | Community (Adult, All Genders) | To investigate the safety, clinical, and neuroimaging outcomes of DBS of the subcallosal cingulate in a group of patients during 12 months of active stimulation. | Quasi-experimental (intervention) | Primary outcomes: safety and acceptability of the procedure. Secondary outcomes: BMI, mood, anxiety, affective regulation, and AN-specific behaviours at 12 months after surgery, changes in neural circuitry (measured with PET imaging). |
| Maguire et al., 2014 | Worldwide | 6 | Mixed (All Genders) | To undertake a targeted summary of the existing literature on OT research and look at the relationship between Oxytocin and Anorexia | Review (Systematic) | Oxytocin level |
| Manos et al., 2018 | USA | 24 | Inpatient (Young People, Women) | To evaluate the effectiveness and tolerability of omega-3 polyunsaturated fatty acid (PUFA) supplementation for treatment of trait anxiety among adolescent females with restrictive anorexia nervosa (AN). | RCT | Trait of anxiety |
| Marquez et al., 2021 | Worldwide | N/A | Community (Adult, All Genders) | To synthesise the best available clinical evidence on the efficacy and safety of second-generation antidepressants and antipsychotics in patients with anorexia nervosa. | Review (Systematic) | Weight (BMI), psychopathological entities, safety |
| Marzola et al., 2015 | Italy | 75 | Inpatient (Adult, All Genders) | To garner preliminary data on the real-world use of olanzapine and aripiprazole as augmentation agents of Selective Serotonin Reuptake Inhibitors (SSRIs) in adult inpatients affected by AN | RCT | Eating symptomatology, and both general and eating psychopathology using: Hamilton Rating Scale for Anxiety (HAM-A), Hamilton Rating Scale for Depression (HAM-D, and Yale-Brown-Cornell Eating Disorders Scale (YBC-EDS) |
| Matsui et al., 2021 | Worldwide | 49 | Community (Adult, All Genders) | To determine the efficacy of ramelteon in treating abnormal eating behaviour in patients with sleep-related eating disorder and/or night eating syndrome. | Review (Other) | Frequency of night time eating, sleep-wake rhythm dysregulation |
| McElory et al., 2011 | USA | 40 | Outpatient (Adult, All Genders) | To assess preliminarily the effectiveness of acamprosate in BED. | RCT | Primary: binge eating episode frequency. Secondary: weekly frequencies of binge days, weight, BMI, CGI-S and CGI-I, YBOCS-BE, Food craving inventory (FCI), Three Factor Eating Questionnaire (TFEQ), Montgomry Asberg Depression Rating Scale (MADRS), medical outcomes study 12-item short form health survey (SF-12). |
| McElroy et al., 2013 | USA | 62 | Outpatient (Adult, All Genders) | To assess preliminarily the effectiveness of a novel opioid antagonist, ALKS-33, in BED. | RCT | Binge eating (weekly binge frequency and days), Body weight, Eating pathology, weight, BMI, waist circumference, clinical assessment (YBOCS-BE, CGI-S, Eating inventory (EI), FCI and BDI) |
| McElroy et al., 2014 | USA | 260 | Community (Adult, All Genders) | To examine the effects of LDX on a range of behavioural and psychological features in individuals with BED by describing the effects of LDX on measures of binge eating behaviour and obsessive–compulsive and impulsive features beyond those previously reported for binge eating frequency. | RCT | Primary outcome: binge eating frequency. Secondary outcome: binge eating behaviours, obsessive-compulsive binge-related and impulsive features. |
| McElroy et al., 2015 | Worldwide | N/A | N/A | To review the WFSBP guidelines and the literature on the pharmacotherapy of eating disorders published since then with a focus on randomized, placebo-controlled trials (RCTs) | Review (Critical) | Psychopharmacological treatments for EDs |
| McElroy et al., 2015 | USA | 260 | Outpatient (Adult, All Genders) | To examine the efficacy and safety of lisdexamfetaminedimesylate, a dextroamphetamine prodrug, to treat moderate to severe BED. | RCT | Binge eating; Binge Eating Scale; BIS-11 (Barratt Impulsiveness Scale, version 11); early termination; HAM-A (Hamilton Anxiety Rating Scale); MADRS (Montgomery-Åsberg Depression Rating Scale); SF-12 (12-Item Short Form Health Survey, version 2); TFEQ (Three-Factor Eating Questionnaire); Y-BOCS-BE (Yale- Brown Obsessive Compulsive Scale modified for BE) |
| McElroy et al., 2015 | USA | 60 | Community (Adult, All Genders) | To evaluate the efficacy, tolerability, and safety of armodafinil in the treatment of binge eating disorder (BED) | RCT | BE days/week, BE episodes/week, CGI-S and CGI-I scores, YBOCS-BE Scores, EI scores, BFI scores, IDA scores, Beck anxiety inventory (BAI) scores, weight, BMI |
| McElroy et al., 2016 | USA | Study 1 = 383, Study 2 = 390 (Total = 773) | Community (Adult, All Genders) | To evaluate the efficacy of LDX vs Placebo in BED. | RCT | Change in baseline BE days/week, CGI-I, proportion of participants with 4-week binge eating cessation at week 12/ET, Y-BOCS-BE score, weight change |
| McElroy et al., 2017 | USA | 724 | Community (Adult, All Genders) | To examine the time course of efficacy-related endpoints for lisdexamfetamine dimesylate (LDX) versus placebo in adults with protocol-defined moderate to severe binge-eating disorder (BED). | RCT | Self-reports (binge eating days/week, binge eating episodes/week, and 1-week binge eating response), CGI-I (from the perspective of BED symptoms), Y-BOCS-BE (at weeks 4, 8, 12), Body weight. |
| McElroy et al., 2020 | USA | 315 | Community (Adult, All Genders) | To evaluate the efficacy and safety of dasotraline, a novel dopamine and norepinephrine reuptake inhibitor, in adults with BED. | RCT | Primary outcome: Change in the number of binge-eating days per week. Secondary outcomes: Y-BOCS-BE, percentage of subjects with cessation of binge eating in the final 4 weeks |
| McKnight and Park, 2010 | Worldwide | N/A | Mixed (All Genders) | To evaluate the literature on the use of atypical antipsychotics in AN. | Review (Other) | BMI, anxiety and depression symptoms, ED symptoms |
| Miniati et al., 2016 | Worldwide | N/A | Community (Adult, All Genders) | To summarize evidence from research on psychopharmacological options for adult patients with anorexia nervosa (AN) | Review (Critical) | Effect of psychopharmacological options (antidepressants, antipsychotics, lithium, other) in treating EDs |
| Misra et al., 2014 | USA | 72 | Outpatient and Inpatient (Young adult, women) | To look at impacts of Estrogen replacement on AN's eating attitudes, anxiety and body image | RCT | Anxiety, eating behaviour, body image |
| Mitchell, Roerig and Steffen, 2013 | Worldwide | N/A | Community (Adult, All Genders) | To provide a comprehensive review of pharmacotherapy and other biological treatments for eating disorders. | Review (Critical) | Pharmacological treatment of EDs |
| Mond and Calogero, 2009 | Australia | 286 | Outpatient (Adult, All Genders) | To (1) compare eating disorder patients and healthy women on measures of obligatory exercise, motivation for exercise, and frequency of hard exercise for weight or shape reasons, in order to elucidate the nature of excessive exercise among individuals with eating disorders; (2) compare excessive exercise behaviour between subgroups of eating disorder patients. | Quasi-experimental (intervention) | Self-report measures - Commitment to Exercise Scale, Reasons for Exercise Inventory, Frequency of 'hard exercise for weight and shape reasons' |
| Monge et al., 2015 | USA | 635 | Community (Adolescents, All Genders) | To examine the use of psychopharmacologic medications in adolescents referred for treatment of restrictive ED, potential factors associated with their use, and reported psychiatric comorbidities | Longitudinal (<5yr) | Reported psychopharmacologic medication use |
| Moola et al., 2013 | Worldwide | 10 | Community (Adult, All Genders) | To review literature on the impact of exercise training interventions for patients living with AN. | Review (Systematic) | BMI, eating disorder symptoms, quality of life, psychological wellbeing, compliance to treatment, fitness and strength. |
| Murray et al., 2021 | Worldwide | 20 | Community (Adult, All Genders) | To extend the scope of earlier reviews by (i) systematically assessing all neurosurgical and neuromodulatory treatments for AN, (ii) aggregating novel evidence not included in previous reviews, (iii) offering alternative findings from recent interpretations of the existing data, and (iv) outlining key initiatives for future research. | Review (Systematic) | Weight symptoms, psychological symptoms |
| Nazar et al., 2017 | Worldwide | 24 | Community (Adult, All Genders) | To collate the literature relating to the early response to treatment in eating disorders and conduct a meta-analysis using diagnostic test accuracy methodology to examine the robustness of the early response concept as a predictor of outcome. | Systematic Review/ Meta-Analysis (combined) | Accuracy measures (sensitivity, specificity, positive likelihood ratios and negative likelihood ratios) |
| Ng, Ng and Wong, 2013 | Worldwide | 9 | Community (Adult, All Genders) | To examine the effects of supervised exercise training in patients with anorexia nervosa. | Meta-Analysis | Anthropometric measurements, weight, body fat, cardiovascular fitness, feelings about food and exercise |
| Norris et al., 2011 | Canada | 86 | Outpatient (Young Adult, Women) | To examine assessment and treatment profiles of adolescent patients with anorexia nervosa and eating disorder not otherwise specified who received olanzapine as compared with an untreated matched sample. | Quasi-experimental (intervention) | Drive for thinness, body satisfaction, BMI, Children’s Depression Inventory; Multidimensional Anxiety Scale for Children; ED |
| Nourredine et al., 2020 | Worldwide | 3 | Community (Adult, All Genders) | To assess the efficacy and safety of topiramate in treating binge eating disorder (BED), using a systematic review and meta-analysis of the available randomized clinical trials (RCTs). | Systematic Review/ Meta-Analysis (combined) | Primary outcomes: (1) change in binge frequency (2) change in quality of life (3) change in weight or BMI; Secondary outcomes: (1) change in obsessive–compulsive symptoms and impulsivity outcomes (2) changes in depression (3) treatment retention measured through dropout rates (4) safety features measured using the number of participants reporting serious adverse events or adverse events reported as the cause of a drop out. |
| Nourredine et al., 2021 | Worldwide | 62 | Community (Adult, All Genders) | To fill the research gap by reviewing data on the use of topiramate in addictive and eating disorders, and to assist in defining its role in the management of these disorders. | Review (Systematic) | Treatment schemes, efficacy, and safety features |
| Ostermann et al., 2019 | Worldwide | 8 | Community (Adult, All Genders) | To systematically assess and the effectiveness and safety of yoga in patients with eating disorders. | Review (Systematic) | Primary outcomes: Improvements in disorder-specific or general eating-related symptoms, BMI. Secondary outcome: safety. |
| Pacanowski et al., 2017 | USA | 38 | Inpatient (Adult, All Genders) | To investigate the effect of yoga on mealtime negative affect and eating disorder symptoms, using a randomized, controlled study design, during residential eating disorder treatment. | RCT | Primary outcomes: EAQ, EDE-Q, PANAS, Hamilton Anxiety Scale (HAS). Secondary outcomes: observer ratings of participant's anxiety, scores on measures assessing distress tolerance and ED symptoms. |
| Park, Godier and Cowdrey, 2014 | Worldwide | N/A | Community (Adult, All Genders) | To explore the neuroscientific basis of aberrant reward in AN, with the aim of generating novel hypotheses for translational investigation and elucidate disease mechanisms to inform the development of targeted interventions. | Review (Other) | Neural basis of aberrant reward in AN. |
| Paslakis et al., 2018 | Germany | 50 | Inpatient (adult, women) | To implement estrogen replacement in AN patients, in order to examine its effects upon AN-associated and general psychopathology, neuropsychological performance and concentrations of peptide components of the hypothalamus-pituitary-adrenal (HPA) axis and within appetite-regulating circuits. | RCT | Primary: change in neuro- psychological performance, secondary: a) incidence of treatment-emergent adverse events (AEs) (safety/tolerability) mirrored by the number of AEs (including AEs, adverse reactions [ARs], serious AEs, serious ARs and suspected unexpected serious ARs [SUSARs]), b) changes in psychopathology (EDE-Q, EDI-2, STAI, Patient health questionnaire (PHQ-9), eating disorder quality of life (EDQOL)) mirrored by changes in sum scores, c) changes in cortisol mirrored by changes in plasma cortisol levels during a dexamethasone suppression test as well as changes in plasma concentrations of the appetite-regulating peptides ghrelin, leptin, insulin and glucose during an OGTT and d) changes in antidepressant medication mirrored by changes in their use. |
| Pataky et al., 2013 | Italy | 289 | Community (Adult, All Genders) | To evaluate the effects of rimonabant on body weight in obese patients with binge eating disorders. | RCT | BE scale total score, Body weight |
| Potes et al., 2021 | Worldwide | 11 | Community (Adult, All Genders) | To assess the use of DBS in treating eating disorders (EDs) to determine its utility and the extent of adverse effects. | Review (Systematic) | BMI, therapeutic outcomes (based on reports of successful treatment of AN symptom, attenuation of a normal menstrual cycle, decrease in binge behaviour, performance on neuropsychological tests, and metabolic rate changes) |
| Powers, Klabunde and Kaye, 2012 | USA | 21 | College (Adult, All Genders) | To (1) determine the effect of quetiapine compared with placebo in terms of reducing core eating disorder symptoms on the Yale–Brown–Cornell Eating Disorder Scale (YBC-EDS) and the Eating Disorder Inventory-2 (EDI-2); (2) determine if quetiapine is superior to placebo in reducing anxiety, depression and obsessionality assessed with the State Trait Anxiety Inventory (STAI), Hamilton Depression Rating Scale (HAM D) and Yale–Brown Obsessive Compulsive Scale, respectively; Other: to determine if quetiapine is superior to placebo in terms of weight gain | RCT | Weight, vital signs, assessment of symptoms, HAM D, Yale–Brown Obsessive Compulsive Scale, YBC-EDS, The Positive and Negative Syndrome Scale (PANSS), EDI-2, STAI. |
| Quilty et al., 2013 | USA | 49 | Outpatient (Adult, Women) | To evaluate the efficacy of psychostimulant medication versus current best practices in the treatment of BED symptoms, in a randomized trial of methylphenidate versus CBT for BED. | RCT | Objective binge episode frequency; Subjective binge episode frequency; BMI; Eating Disorder Inventory – Interview; Binge Eating Scale; Quality of Life Inventory |
| Rachid, 2018 | Worldwide | N/A | Community (Adult, All Genders) | To review the literature on the safety and efficacy of repetitive transcranial magnetic stimulation and its modified stimulation paradigms (deep TMS and theta-burst stimulation) as a treatment for eating disorders, and discuss results and future directions for research in this growing area of attention. | Review (Other) | Safety and efficacy of TMS for treating EDs (considered psychological and physiological responses) |
| Ramacciotti et al., 2013 | Worldwide | N/A | N/A | To address the state of the art concerning the treatment of BED | Review (Narrative) | Binge eating and body weight |
| Reas and Grilo, 2014 | Worldwide | 26 | N/A | To evaluate the controlled treatment studies of pharmacotherapy for binge eating disorder (BED) | Review (Systematic) | Binge eating frequency, remission, weight loss, CGI scores, objective bulimic episodes (OBE) frequency, |
| Reas, & Grilo, 2021 | Worldwide | N/A | Community (Adult, All Genders) | To provide a brief state-of-the-art review of randomized controlled trials (RCTs) for combined psychological and pharmacological treatment of AN, BN, and BED. | Review (Critical) | Efficacy of combined treatments for AN, BN and BED. For AN: percentages of ideal body weight over time, psychological functioning, weight restoration, AN severity rating, depression BMI, obsessional symptoms, compulsive symptoms, depressive symptoms. For BN: purging frequency, binge-eating frequency, depression, abstinence/remission. For BED: percent binge abstinent, binge-eating frequency, weight loss, depression, eating pathology. |
| Robert et al., 2015 | Malaysia | 44 | Community (Adult, All Genders) | To examine the efficacy of liraglutide on appetite and plasma ghrelin in non-diabetic obese participants with BE. | RCT | BES, ghrelin levels, other anthropometric variables |
| Rosager, Moller, & Sjogren, 2021 | Worldwide | 4 | Community (Adult, All Genders) | To review treatment studies with cannabinoids in anorexia nervosa | Review (Systematic) | Effect on weight. Secondary outcomes: affective symptoms, eating disorder related personality changes, physical activity (PA) and blood pressure. |
| Russell et al., 2018 | Australia | 41 | Inpatient (young adult, women) | To ascertain whether repeated doses of IN-OT enhance treatment outcomes in AN | RCT | Weight gain, EDE eating scale, social anxiety, morning plasma OT levels, anticipation of an afternoon snack |
| Sala et al., 2016 | USA | 24 | Community (Adult, All Genders) | To evaluate whether chromium picolinate (CrPic) supplementation improves glucose regulation in overweight individuals with binge-eating disorder (BED) | RCT | Change in glucose, Change in Insulin, Change in ISI |
| Sauchelli et al., 2015 | Spain | 88 | Community (Adult, Women) | To examine objectively measured physical activity in this clinical population and how it might be related to a partial hospitalization therapy response, after considering potential confounders | Quasi-experimental (intervention) | Physical activity, daytime PA, time in MVPA, high vs low PA levels, BMI and body composition, depression symptoms. |
| Schlegel et al., 2015 | Germany | 36 | Outpatient (Adult, All Genders) | To develop a sport therapy program for ED outpatients aiming (a) to reduce dysfunctional attitudes toward physical exercise as well as unhealthy exercise behaviour and (b) to use positive effects of physical activity on body experience, emotion regulation and self-esteem. | Quasi-experimental (intervention) | Primary outcome: Commitment to Exercise Scale (CES) score. Secondary outcomes: EDI-2 scale drive for thinness, and body dissatisfaction. Other: eating pathology (EDE-Q), quality of life (SF-12), body weight, BMI, amount of physical activity. |
| Shih et al., 2017 | N/A | N/A | N/A | To review the clinical benefits PUFA treatments exert in other psychiatric diseases, on weight and appetite regulation, and for resolution of inflammation, all of which are relevant in the disease course and outcome of AN | Review (Narrative) | Benefits of PUFA |
| Sysko et al., 2010 | Worldwide | 785 | Community (Adult, All Genders) | To examine the relationship between initial and later response to fluoxetine | Modelling (Statistical) | Treatment efficacy, compliance, ED symptoms |
| Val-Laillet et al., 2015 | Worldwide | N/A | Community (Adult, All Genders) | To (1) discuss the possibility to identify new biological markers of brain functions; (2) highlight the potential of neuroimaging and neuromodulation in individualized medicine; (3) To introduce the ethical questions that are concomitant to the emergence of new neuromodulation therapies. | Review (Other) | Neuroimaging and neuromodulation approaches are assessed in terms of technical challenges, applicability and ethics |
| Vander Wal et al., 2012 | England | 40 | Community (Adult, All Genders) | To examine the short-term effects of escitalopram on symptoms of NES in a randomized controlled clinical trial | RCT | Change in night eating questionnaire (NEQ) |
| Vander Wal et al., 2015 | USA | 44 | Community (Adult, All Genders) | To test a novel, brief intervention for NES and to evaluate the feasibility of recruitment, randomization, retention, and intervention implementation | RCT | NEQ scores, BDI scores, BAI scores, perceived stress scale (PSS) scores, night eating diagnostic questionanire (NEDQ) symptoms, Sleep logs |
| Vander Wal, 2012 | Worldwide | N/A | Community (Adult, All Genders) | To provide an introduction to NES, including diagnosis, clinical presentation, assessment, comorbidities, clinical implications, and pharmacological and psychological treatment approaches | Review (Critical) | Information reviewed includes diagnosis, clinical presentation, assessment, comorbidities, clinical implications, and pharmacological and psychological treatment approaches |
| Ward and Citrome, 2018 | Worldwide | N/A | N/A | To describe the chemistry and pharmacology of LDX, as well as the clinical trials investigating the efficacy and safety of this medication for the management of BED. | Review (Narrative) | Absorption, distribution, excretion, metabolism, pharmacogenetics, efficacy |
| Watson et al., 2019 | Worldwide | 33 | Community (Adult, All Genders) | To combine data from the Anorexia Nervosa Genetics Initiative (ANGI) and the Eating Disorders Working Group of the Psychiatric Genomics Consortium (PGC-ED) and conduct a genome-wide association study, identifying eight significant loci. | Meta-Analysis | Analysis of chromosome X, Female-only GWAS, Distance- and LD-based clumping, Conditional and joint analyses, multi-trait based conditional and joint analyses, eQTL and chromosome conformation capture (Hi-C) interactions, SNP-based heritability estimation, Polygenic risk scoring for within-trait predictions, Anorexia nervosa subtype analysis, Genetic correlations in the cross-trait analysis, GWAS of related traits, GSMR analyses, Gene-wise analysis, Partitioned heritability. |
| White and Grilo, 2013 | USA | 61 | Community (Adult, Women) | To perform a randomized placebo-controlled trial to evaluate the short-term efficacy of bupropion for the treatment of BED in overweight and obese women. | RCT | Primary: binge eating frequency (self-report diaries), percent BMI loss. Secondary: eating disorder psychopathology, food craving (FCI), depression levels (BDI). |
| Yu et al., 2011 | USA | 122 | Community (Adult, All Genders) | To examine maintenance of recovery following treatment in an adult anorexia nervosa (AN) population | RCT | The primary outcomes were weight and the global scores from the Eating Disorder Examination (EDE) separately and combined. |
